# Gender sensitivity of the COVID-19 mental health research in Europe: a scoping review

**DOI:** 10.1186/s12939-024-02286-1

**Published:** 2024-10-10

**Authors:** Mayte López-Atanes, Margarita Sáenz-Herrero, Nele Zach, Meret Lakeberg, Asier Ugedo, Elisa Fraile-García, Leire Erkoreka, Rafael Segarra, Ingo Schäfer, Tilman Brand

**Affiliations:** 1 Center for Interdisciplinary Addiction Research, Department of Psychiatry and Psychotherapy, University Medical Center Hamburg-Eppendorf, Hamburg, Germany; 2 Department of Psychiatry and Psychotherapy of the University Medical Center Hamburg-Eppendorf, Hamburg, Germany; 3 University of the Basque Country UPV/EHU, Leioa, Spain; 4 Cruces University Hospital, Barakaldo, Spain; 5 Biocruces-Bizkaia Health Research Institute, Barakaldo, Spain; 6 Leibniz Institute for Prevention Research and Epidemiology - BIPS, Bremen, Germany; 7 Psychiatry Service, Barrualde Galdakao Integratet Health Organization, Galdakao, Spain; 8 CIBERSAM, ISCIII, Madrid, Spain

## Abstract

**Background:**

The integration of sex and gender aspects into the research process has been recognized as crucial to the generation of valid data. During the coronavirus pandemic, a great deal of research addressed the mental state of hospital staff, as they constituted a population at risk for infection and distress. However, it is still unknown how the gender dimension was included. We aimed to appraise and measure qualitatively the extent of gender sensitivity.

**Methods:**

In this scoping review, we searched MEDLINE, EMBASE, CINAHL PsycINFO and Social Sciences Citation Index (SSCI) from database inception to November 11, 2021. All quantitative studies with primary data published in English, German, or Spanish and based in the European Union were selected. Included studies had to have assessed the mental health of hospital staff using validated psychometric scales for depression, anxiety, PTSD symptoms, distress, suicidal behavior, insomnia, substance abuse or aggressive behavior. Two independent reviewers applied eligibility criteria to each title/abstract reviewed, to the full text of the article, and performed the data extraction. A gender sensitivity assessment tool was developed and validated, consisting of 18 items followed by a final qualitative assessment. Two independent reviewers assessed the gender dimension of each included article.

**Results:**

Three thousand one hundred twelve studies were identified, of which 72 were included in the analysis. The most common design was cross-sectional (75.0%) and most of them were conducted in Italy (31.9%). Among the results, only one study assessed suicidal behaviors and none substance abuse disorders or aggressive behaviors. Sex and gender were used erroneously in 83.3% of the studies, and only one study described how the gender of the participants was determined. Most articles (71.8%) did not include sex/gender in the literature review and did not discuss sex/gender-related findings with a gender theoretical background (86.1%). In the analysis, 37.5% provided sex/gender disaggregated data, but only 3 studies performed advanced modeling statistics, such as interaction analysis. In the overall assessment, 3 papers were rated as good in terms of gender sensitivity, and the rest as fair (16.7%) and poor (79.2%). Three papers were identified in which gender stereotypes were present in explaining the results. None of the papers analyzed the results of non-binary individuals.

**Conclusions:**

Studies on the mental health of hospital staff during the pandemic did not adequately integrate the gender dimension, despite the institutional commitment of the European Union and the gendered effect of the pandemic. In the development of future mental health interventions for this population, the use and generalizability of current evidence should be done cautiously.

**Supplementary Information:**

The online version contains supplementary material available at 10.1186/s12939-024-02286-1.

## Introduction

There is rising awareness of the need to integrate sex and gender in health research to increase the validity and generalizability of study findings. Gender is a multidimensional variable describing identity, social norms, and relations between individuals, while sex is a biological construct encompassing the biological characteristics enabling reproduction [1, 2]. Although traditionally conceptualized as two separate constructs, sex and gender are interrelated, and the binary distinction between women/men and female/male does not capture all the existing variability. In accordance with other authors, we used the shortened version sex/gender. This highlights that even being distinct concepts, there are potential interrelations between biological and sociocultural aspects of being a man, a woman, or a sex/gender diverse person [3]. Both sex and gender can influence the presentation of diseases, the diagnosis, and even the access to treatment and available support [2, 4–7]. In the case of mental health disorders, there are clear epidemiological differences regarding sex/gender, although it remains unclear to what extent the differences are due to biological or social factors. In general, externalizing disorders, such as violent behavior or substance abuse, are more often reported among men, while the majority of patients with internalizing syndromes like depression and anxiety are women [8]. This pattern was also observed during the COVID-19 pandemic: most studies revealed that women presented more depressive and anxiety symptoms than men, and this was particularly true in the healthcare sector [9–13]. Front-line medical staff had the highest levels of distress and perception of life threat, as hospitals were one of the main settings for infection during the first waves.

Gender equity has been acknowledged as a relevant transversal issue in European Union (EU) policymaking since the late 1990ies, when the concept of gender mainstreaming was introduced [14]. Sex/gender sensitivity can be conceptualized as the consideration of sex/gender aspects in all the steps of the research process [15]. Additionally, it strives to provide equal participation of women and men in scientific work and consider non-binary individuals [16]. Even if the primary research question of a health study does not focus on sex or gender, sensitivity towards it is warranted because all cells are sexed, and all bodies are gendered [17, 18]. In the last decades, several countries and institutions developed guidelines and recommendations on how to achieve sex/gender sensitivity, but the implementation has been slow [19–21]. The EU, in particular, published a guideline in 2012 on how to include gender sensitivity in research [22], but also high-impact journals showed their commitment to the appropriate use of sex/gender and provision with disaggregated data [23]. Additionally, the SAGER guidelines provide orientation for journal editors on how to evaluate the inclusion of gender in a paper. Parallelly, individual studies provided examples of good practices respecting gender or checked the current status of the integration of sex and gender in research proposals [3, 24–26]. However, o date, there is no tool to adequately assess the gender sensitivity of an article.

A lack of sex/gender sensitivity can lead to biased research results, delayed diagnosis or undertreatment [27, 28]. In terms of studies on the psychological impact of the pandemic on healthcare workers, evidence as to how sex/gender has been integrated into research is almost nonexistent. Although an emerging body of literature demonstrated the gendered impact of the pandemic on this population [11, 29, 30], to date, no study has assessed how sex and gender have been included globally throughout the research process. In this context, we set out to assess the extent of gender sensitivity in studies on the psychological impact of hospital staff during the COVID-19 pandemic. Given the strong EU commitment to sex/gender sensitivity in research, we focused specifically on EU studies and assessed how and to what extent studies included these variables.

### Review question and objectives

Is current literature about the psychological impact of the coronavirus on healthcare workers gender-sensitive? Specifically to:

- How sex/gender is assessed in the articles.
- How are the results and conclusions presented with respect of sex/gender.
- Potential gender bias in the interpretation of results.

## Materials and methods

We conducted a scoping review of peer-reviewed literature in line with the PRISMA-ScR guidelines. The respective protocol was registered in the Open Science Framework (OSF) 10.17605/OSF.IO/XBU5A. We chose a scoping review methodology because our objective was not to answer a specific research question but to do a comprehensive mapping of the published studies.

### Search strategy and selection criteria

We searched (from database inception to 11 November 2021) MEDLINE via OvidSP, EMBASE via OvidSP, CINAHL via EBSCO, PsycINFO via OvidSP, Social Sciences Citation Index (SSCI) via web of Science. The search terms were developed iteratively by the research team including a professional librarian and included three sets of key terms (Healthcare workers, Mental health, and Hospital) combined with Boolean logic to search for relevant papers. The complete search strategy for each database is provided in the supplemental material.

### Study selection

To meet the inclusion criteria, articles had to: 1) be peer-reviewed and use quantitative methods; 2) use validated psychometric tests of depression, anxiety, distress/stress, substance use/abuse, suicidal ideation, insomnia, posttraumatic stress disorder (PTSD), or quality of life; 3) focus on hospital-based healthcare workers; 4) be conducted in the European Union. We excluded non-peer-reviewed publications, populations apart from hospital healthcare workers, studies that did not address the impact of the COVID-19 pandemic, non-European studies, and studies published in another language than English, Spanish, or German. The identified studies were stored, deduplicated, and later imported into the software Rayyan for screening by two independent reviewers of titles, abstracts, and full-text articles against the eligibility criteria. We resolved any disagreement by consensus.

### Data extraction and analysis

Data were extracted from included studies using a pilot extraction form in the Google Forms platform form. It included study characteristics (e.g., design, sample, location), sex/gender of first and last author, outcome, results, and the items in the assessment tool. Data extraction for each included article was performed independently by two reviewers (MLA as first reviewer, NZ, AU and ML as second reviewer).

We first performed a bibliography search to find any instrument to appraise the sex/gender sensitivity of a study. As we did not find any validated appraisal tool suitable for our study, we selected critical items from available instruments [15, 22, 31–36]. We primarily followed the structure of the SAGER guidelines, designed to guide the report of sex/gender in research, and developed the appraisal tool questions based on this structure. The items of the tool were further developed and revised by two senior researchers with expertise in gender studies (TB and MS) and a PhD student in gender studies (MLA). Each item was redefined until consensus was reached. We developed 18 items divided into five sections, followed by a rating of each section and the overall rating using an ordinal scale with the items "excellent", "good", "fair" and "poor”. Subsequently, we tested the inter-rater reliability. Initially, we conducted a pilot test with 10 randomly selected items and redefined the items with the lowest inter-rater agreement values. In the second step, we scored 18 articles that mentioned sex and/or gender in the title or abstract. A researcher with expertise in gender studies (MLA) and a second rater (EF) participated in this process. Most of the values were in an acceptable range. The kappa for the item "overall rating" was 0.577, showing moderate agreement among the raters. Supplementary Appendix provides the appraisal tools and the κ scores for inter-rater agreement.

## Results

**Fig. 1:**
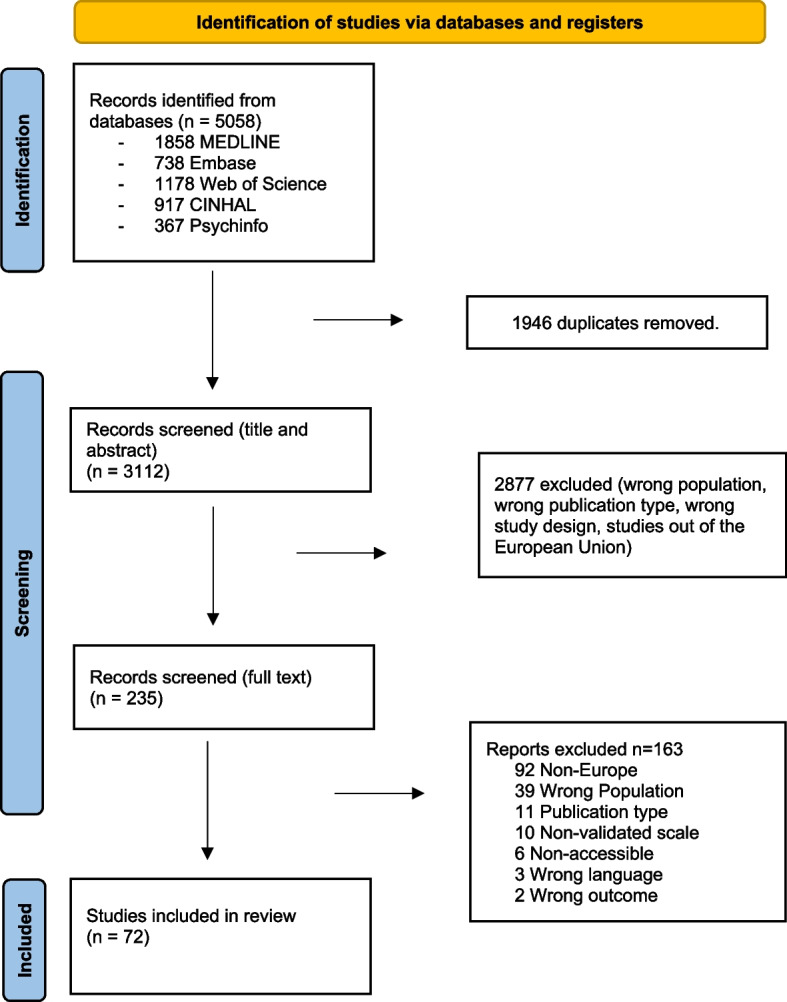
Flowchart of the included studies

We identified 3112 articles after the removal of duplicates (Fig. 1). We assessed the full texts of 235 articles for eligibility. Of these, 125 studies did not examine the population under study (other populations than healthcare workers or healthcare workers from outside the EU); 17 articles did not contain original data; 12 did not examine the required outcomes; 4 were not in English, German, or Spanish, 3 did not have a quantitative study design and 3 were background articles. We included 72 independent studies in the analysis [30, 37–107] (Fig. 1). The complete list and characteristics of the included studies is provided in Table 1.

**Table 1:** list of included studies

	**Country**	**Study design**	**% of women**	**Correct Use of sex/gender**	**Sex/gender in the literature review**	**Sex/Gender in the objectives**	**Non-binary category in data collection**	**Disaggregated outcome data**	**Sex/Gender in statistics**	**Gender theory in the discussion**	**Gender stereotypes**	**Overall Appraisal**
Bettinsoli et al. (2020) [74]	Italy	Cross-Sectional	40.0	No	Yes	Yes	No	Only one of the outcomes	Factor in the regression Interaction analysis	Yes. Differences in reported distress may be explained by gender roles and expectations	No	Good
López-Atanes et al. (2021) [30]	Spain	Cross-sectional	74.6	No	Yes	Yes	No	Yes	Group comparison Factor in regression Interaction analysis	Yes. Mention to the double burden in women and its relation to mental health	No	Good
Moreno-Mulet et al. (2021) [97]	Spain	Mixed-methods	81.1	No	Yes	Yes	No	Yes	Group comparison	Yes. Mention to gender roles and expectations	No	Good
Collantoni et al. (2021) [41]	Italy	Cross-Sectional	75.8	No	Yes	No	No	Yes	Factor in the regression	Yes; only mention to women`s caregiver role	No	Fair
Roberts et al. (2021) [54]	International	Prospective Cohort	51.0	No	No	No	Yes	No	Factor in the regression	No	No	Fair
Erquicia et al. (2020) [64]	Spain	Cross-sectional	73.6	No	Yes	No	No	Yes	Factor in regression Interaction analysis	No	No	Fair
Carmassi et al. (2021) [68]	Italy	Cross-Sectional	68.3	No	Yes	No	No	Yes	Factor in the regression	No	No	Fair
Fattori et al. (2021) [75]	Italy	Longitudinal	46.0	No	No	No	No	Yes	Factor in the regression	No	No	Fair
Carmassi et al. (2021) [83]	Italy	Cross-sectional	56.8	No	Yes	Yes	No	Yes	Factor in regression Interaction analysis	No	No	Fair
Forner-Puntonet et al. (2021) [87]	Spain	Intervention study	76.6	No	Yes	No	No	No	Group comparison	Yes. Mention to the gender perspective in mental health research	No	Fair
Gago-Valiente et al. (2021) [88]	Spain	Cross- sectional	84.9	No	No	No	No	Yes	Group comparison	Yes. Mention to gender related variables and their influence in mental health	No	Fair
Malinowska-Lipién et al. (2021) [95]	Poland	Cross-Sectional	97.0	Yes	No	No	No	Yes	Group comparison	Yes. Gender segregation of labor in the healthcare sector may explain poorer mental health in professions such as nursing	No	Fair
Weseman et al. (2021) [103]	Germany	Cross-sectional	67.0	No	Yes	Yes	No	No	Regression analysis	Yes. Mention to the double burden in women and its relation to mental health	No	Fair
Morawa et al. (2021) [107]	Germany	Cross-sectional	74.8	No	Yes	No	Yes	No	Factor in the regression	No	No	Fair
Mortier et al. (2020) [105]	Spain	Prospective cohort	77.3	No	No	No	Yes	Yes	Group comparison Factor in the regression	No	No	Fair
Aguglia et al. (2021) [37]	Italy	Cross-sectional	67.6	No	No	No	No	No	Group comparison	No	No	Poor
Ali et al. (2020) [38]	Ireland	Prospective cohort	69.0	No	No	No	No	No	FALTA	No	No	Poor
Beneria et al. (2020) [39]	Spain	Cross-sectional	70.0	No	No	No	No	No	Group comparison Factor in Regression	No	Yes	Poor
Bidzan et al. (2020) [40]	Poland	Cross-sectional	74.4	No	Yes	No	No	No	Group comparison Factor in regression	No	No	Poor
Di Giuseppe et al. (2021) [42]	Italy	Cross-sectional	62.0	No	Yes	No	No	No	Group comparison Factor in regression	No	No	Poor
Dionisi et al. (2021) [43]	Italy	Longitudinal	49.0	No	No	No	No	No	Not analyzed	No	No	Poor
Ghio et al. (2021) [44]	Italy	Cross-sectional	76.0	No	Yes	No	No	No	Factor in regression	No	No	Poor
Giusti et al. (2020) [45]	Italy	Cross-sectional	62.6	No	No	No	No	No	Factor in regression	No	No	Poor
Haravuori et al. (2020) [46]	Finland	Prospective cohort	87.5	No	No	No	Yes, “other or prefer not to say”	No	Factor in regression	No	No	Poor
Lamiani et al. (2021) [47]	Italy	Cross-sectional	80.0	No	No	No	No	No	Factor in regression	No	No	Poor
Lasalvia et al. (2021) [48]	Italy	Cross-sectional	75.3	No	No	No	No	Yes	Group comparison Factor in regression	No	No	Poor
Lucas et al. (2021) [49]	France	Cross-sectional	79.2	No	Yes	No	No	No	Factor in regression	No	No	Poor
Magnavita et al. (2021) [50]	Italy	Prospective cohort	51.7	No	No	No	No	No	Factor in regression	No	No	Poor
Man et al. (2020) [51]	Romania	Cross-sectional	88.7	No	No	No	No	No	Group comparison	No	No	Poor
Matarazzo et al. (2021) [52]	Italy	Cross-sectional	80.2	No	No	No	No	Yes	Group comparison Factor in regression	No	No	Poor
Pappa et al. (2021) [53]	International	Cross-sectional	68.8	No	Yes	No	No	Yes	Group comparison Factor in regression	No	No	Poor
Secosan et al. (2020) [55]	Romania	Cross-Sectional	64.3	No	No	No	No	No	Not analyzed	No	No	Poor
Secosan et al. (2020) [56]	Romania	Cross-Sectional	64.3	No	No	No	No	No	Not analyzed	No	No	Poor
Stocchetti et al. (2021) [57]	Italy	Cross-sectional	59.0	No	No	No	No	No	Group comparison Factor in regression	No	No	Poor
Tiete et al. (2021) [58]	Belgium	Mixed-Methods	78.4	No	No	No	No	Yes	Group comparison Factor in regression	No	No	Poor
Tselebis et al. (2020) [59]	Greece	Cross-sectional	80.0	No	No	No	No	Yes	Group comparison	No	No	Poor
Ungureanu et al. (2020) [60]	Romania	Cross-sectional	59.4	No	No	No	No	No	Group comparison	No	No	Poor
Van der Goot et al. (2021) [61]	Netherlands	Mixed-Methods	78.3	Yes	No	No	No	No	Not analyzed	No	No	Poor
Zerbini et al. (2020) [62]	Germany	Cross-sectional	70.0	No	No	No	No	No	Not analyzed	No	No	Poor
Altmayer et al. (2020) [63]	France	Cross-sectional	78.0	Yes	No	No	No	No	Not analyzed	No	No	Poor
Marcomini et al. (2021) [65]	Italy	Cross-sectional	76.3	No	Yes	No	No	No	Factor in regression	No	No	Poor
Roberts et al. (2021) [66]	International	Cross-sectional	49.5	No	No	No	Yes	No	Group comparison	No	No	Poor
Azoulay et al. (2021) [67]	France	Cross-sectional	67.5	No	No	No	No	No	Factor in regression	No	No	Poor
Mattila et al. (2021) [70]	Finnland	Cross-sectional	87.0	No	No	No	No	Yes	Group comparison Factor in regression	No	No	Poor
Simonetti et al. (2021) [71]	Italy	Cross-sectional	66.0	No	Yes	No	No	Yes	Group comparison Factor in regression	No	Yes	Poor
Azoulay et al. (2020) [72]	France	Cross-sectional	71.0	Yes	Yes	No	No	No	Group comparison Factor in regression	No	No	Poor
Salopek-Žiha et al. (2020) [73]	Croatia	Cross-Sectional	Not reported	No	No	No	No	No	Not analyzed	No	No	Poor
Laurent et al. (2021) [76]	France	Cross-sectional	72.6	No	No	No	No	No	Group comparison Factor in the analysis	Yes. Mention to the double burden of women	No	Poor
González-Plaza et al. (2021) [77]	Spain	Cross-sectional	87.0	No	No	No	No	Yes	Not analyzed	No	No	Poor
Heesakkers et al. (2021) [78]	Netherlands	Cross-sectional	73.8	No	No	No	No	No	Factor in analysis	No	No	Poor
Fiabane et al. (2021) [69]	Italy	Cross-sectional	68.2	No	Yes	No	No	Yes	Group comparison Factor in regression	No, but mention to the higher proportion of women in the nursing profession	No	Poor
Hesselink et al. (2021) [79]	Netherlands	Cross-sectional	72.9	No	No	No	No	No	No	No	No	Poor
Abuye et al. (2021) [80]	Spain	Cross-sectional	81.9	No	No	No	No	No	Group comparison	No	No	Poor
Azoulay et al. (2020) [ 81]	International	Cross-sectional	34.2	No	No	No	No	No	Factor in the regression	No	No	Poor
Caillet et al. (2020) [82]	France	Cross-sectional	75.0	No	No	No	No	Yes	Group comparison Factor in the regression	No	No	Poor
Costa et al. (2021) [84]	Italy	Cross-sectional	52.9	No	No	No	No	No	Group comparison Factor in the regression	No	No	Poor
Diomidus et al. (2020) [85]	Greece	Cross-sectional	71.3	No	Yes	No	No	No	No	No	No	Poor
Fari et al. (2021) [86]	Italy	Retrospective cohort	67.6	No	No	No	No	No	Factor in the regression	No	No	Poor
Ilias et al. (2021) [89]	Greece	Cross-sectional	77.0	No	No	No	No	No	Factor in the regression	No	No	Poor
Kapetanos et al. (2021) [90]	Cyprus	Cross-Sectional	80.0	No	No	No	No	No	Factor in the regression	No	No	Poor
Laukkala et al. (2021) [91]	Finland	Prospective cohort	89.0	Yes	No	No	No	No	Group comparison Factor in the regression	No	No	Poor
Leira-Sanmartin et al. (2021) [92]	Spain	Cross-sectional	79.1	No	No	No	No	Yes	Group comparison Factor in the regression	No	Yes	Poor
Magnavita et al. (2021) [93]	Italy	Cross-sectional	61.2	No	No	No	No	No	Group comparison Factor in the regression	No	No	Poor
Magnavita et al. (2020) [94]	Italy	Cross-sectional	52.2	No	No	No	No	No	Group comparison Factor in the regression	No	No	Poor
Moreno-Jiménez et al. (2021) [96]	Spain	Cross-sectional	78.7	No	No	No	No	Yes	Factor in the regression	No	No	Poor
Nijland et al. (2021) [98]	Netherlands	Intervention Study	83.0	No	No	No	No	Yes	Group comparison	No	No	Poor
Peñacoba et al. (2021) [99]	Spain	Cross-sectional	84.8	Yes	No	No	No	Yes	Group comparison Factor in regression not known	No	No	Poor
Schmid et al. (2021) [100]	Germany	Longitudinal	44.8	No	No	No	No	No	Group comparison Factor in the regression	No	No	Poor
Singh et al. (2020) [101]	Sweden	Mixed methods	100%	Not applicable	No	No	No	No	No	No	No	Poor
Sangrá et al. (2021) [102]	Spain	Cross-Sectional	84.8	No	No	No	No	Yes	Group Comparison Factor in the regression	No	No	Poor
Vanni et al. (2020) [104]	Italy	Cross-sectional	65.2	No	No	No	No	Yes	Group comparison	No	No	Poor
Van Steenkiste et al [106]	Belgium	Prospective cohort	81.0	Yes	No	No	No	No	Group comparison	No	No	Poor

### Study characteristics and outcomes

**Fig. 2:**
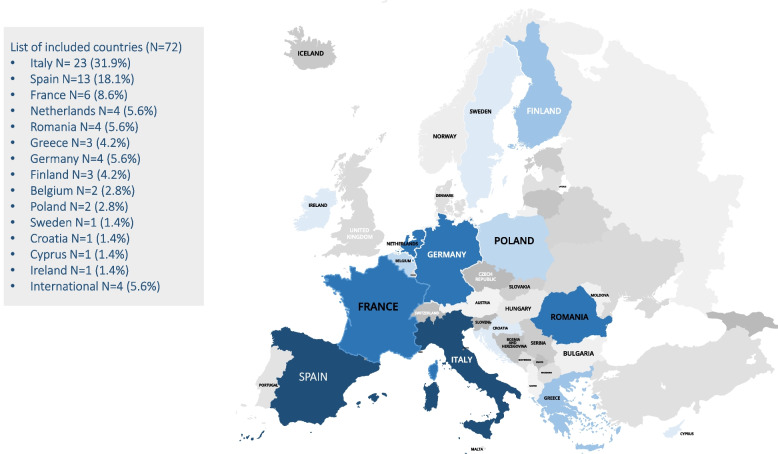
list of included countries. Those in dark blue are the ones with higher proportion of included studies

The most common design was an observational cross-sectional study (54 studies; 75.0%), followed by prospective cohort studies (8 studies, [11.1%]). Most studies were conducted in Italy (31.9%), Spain (18.1%) and France (8.3%), while the remainder came from 11 other EU countries (Fig. 2). The number of participants ranged between 3 and 5440, and the percentage of women was between 34 and 100%. The most frequent outcome variable was depression (49 studies [68.1%]) and anxiety (44 studies [61.1%]). Only one study reported suicidal behavior (1.4%) in the outcome variables, and none evaluated violent or risky behaviors or substance abuse. However, five articles included alcohol or substance use as a sociodemographic variable [44, 51, 81, 84, 108] and one checked for the presence of substance abuse disorders before the pandemic [105]. Overall, the most used psychometric tests were the Impact of Event scale (*n* = 18), the Patient Health Questionnaire (*n* = 15), and the Maslach Burnout Inventory (*n* = 14). Regarding sociodemographic variables that potentially intersect with sex/gender, age, occupation and marital status were present in almost all articles but did not refer to the variables as gender-relevant or used then in a intersectional analysis. However, other variables such as religion [73], migration background [77, 107], and ethnicity [54] were rarely mentioned. In terms of authorship, women constituted 52.8% of the first authors and 41.7% of the last authors. A description of the included studies is provided in the appendix Table 1.

Most of the studies that provided disaggregated data reported gender differences in depressive [30, 41, 48, 64, 75, 92, 109], anxiety [41, 48, 58, 64, 70, 71, 75, 82, 99, 109], stress [30, 38, 52, 59, 64, 96, 109], post-traumatic stress [41, 48, 75, 82, 109] and insomnia [59] symptoms. In general, symptoms were worse among women/females, except two that revealed more depressive symptoms in males/men [69, 88], one of which was rated as fair in terms of gender sensitivity [88]. Regression analyses showed that being a woman was a risk factor for the presentation of stress [30, 39, 41, 42, 52, 64, 76, 102], anxiety [40, 41, 47, 48, 67, 71, 72, 81, 82, 86, 102], depression [30, 41, 47, 48, 52, 67, 72, 74, 81, 92, 102] PTSD [48, 67, 72, 102] and insomnia [71]. Among the three articles rated as good in gender sensitivity, two found statistically significant gender differences in mental health variables, being woman more affected than men [74, 30], and one did not [97]. Advanced modelling techniques were identified in three of the articles. In one of them, age was found to interact with gender: as age increased, symptoms of depression and anxiety decreased in men, whereas they remained stable in women [64]. A second study concluded that stress symptoms, resilience, emotional symptoms, and self-efficacy mediated the influence of the gender variable on psychiatric symptoms [74]. Finally, the third study found that the presence of previous psychiatric history had a greater impact on depressive symptoms in men [30]. Two of this three articles were rated as good in terms of gender sensitivity [74, 30] and one as fair [64].

### Sex/gender sensitivity

A sex/gender sensitivity assessment was performed in each study (Table 2). Seventy-one publications mentioned sex or gender, but only one defined it [30]. A total of 60 articles (83.3%) used the terms sex or gender erroneously or interchangeably. For example, gender was divided into two categories (male and female) that correspond to the sex of individuals, or all terms (sex, gender, male/female, women/men) were used interchangeably throughout the article. None of the papers specifically mentioned the non-binary population. However, five (6.9%) provided the additional label of "other" or "diverse" [46, 54, 66, 105, 107] in the data collection of the sex/gender. In the introduction, most studies did not refer to sex/gender differences or similarities in the literature review (51 studies [71.8%]), and only five (7.0%) mentioned sex/gender in the objectives of the study. In the methods section, one article (1.4%) explained how gender was determined. One paper provided different cut-off values for women in one of the psychometric scales [47], while all the others used the same cut-off values without providing literature to justify it.

**Table 2:** Sex/gender appraisal tool items

	**Frequency (*n* = 72)**		
**General principles**	1. Were sex or gender considered at all?	Yes	71 (98.6%)
		No	1 (1.4%)
	2. Were sex and gender defined?	Yes	1 (1.4%)
		No	71 (98.6%)
	3. Does the paper mention gender categories besides the woman/man binary?	Yes	0 (0%)
		No	72 (100%)
	4. Were the terms sex/gender correctly used in the article?	Yes	8 (11.1%)
		No	60 (83.3%)
		Other/Not applicable	4 (5.6%)
	5. If only one sex/gender was included, was this made clear in the title/abstract?	Other/Not applicable	71 (98.6%)
		No	1 (1.4%)
**Introduction and objectives**	6. Does the literature review include sex/gender differences or similarities?	Yes	21 (29.2%)
		No	51 (70.8%)
	7. Were sex/gender mentioned in the objectives of the study?	Yes	5 (6.9%)
		No	67 (93.1%)
**Methods**	8. Did the authors explain what aspects of sex/gender were analyzed?	Yes	1 (1.4%)
		No	71 (98.6%)
	9. Were other sex/gender categories beyond the men/women binary included in the data collection?	Yes	5 (6.9%)
		No	67 (93.1%)
	10. Are other gender-related variables captured in the study?	Yes	1 (1.4%)
		No	70 (97.2%)
		Other/Not applicable	1 (1.4%)
	11. Did the authors consider the gender sensitivity of the tools used in the study?	Yes	1 (1.4%)
		No	71 (98.6%)
**Analysis and results**	12. Is data presented disaggregated by sex/gender?	Yes	27 (37.5%)
		No	45 (62.5%)
	13. Is there an adequate representation of women and men?	Yes	14 (19.7%)
		No	57 (79.2%)
		Other/not applicable	1 (1.4%)
	14. Was sex/gender appropriately included as a factor a regression analyses?	Yes	47 (65.3%)
		No	23 (31.9%)
		Other/Not applicable	2 (2.8%)
	15. Were sex/gender differences analyzed using advanced modeling techniques (like interaction analysis)?	Yes	3 (4.2%)
		No	68 (94.4%)
		Other/Not applicable	1 (1.4%)
**Discussion**	16. Were the findings reflected concerning sex/gender?	Yes	10 (13.9%)
		No	62 (86.1%)
	17. Were gender stereotypes present in the interpretation of the data?	Yes	4 (5.6%)
		No	68 (94.4%)
	18. Were there any inadequate generalizations in the study?	Yes	1 (1.4%)
		No	69 (95.8)
		Other/Not applicable	2 (2.8%)

In the analysis and reporting of the results, 27 studies (37.5%) disaggregated outcome data in relation to sex/gender studies [30, 38, 41, 48, 53, 58, 59, 64, 68–71, 74, 75, 77, 82, 83, 88, 92, 95–99, 102, 104, 105]. Fourteen studies (19.7%) had an adequate representation of women/females or men/males (measured as a proportion between 40 to 60% or equivalent to the sex/gender ratio in the underlying population) [30, 43, 50, 54, 57, 60, 66, 74, 75, 83, 84, 94, 100, 107]. In contrast, 57 studies had an overrepresentation of one gender, and one study included only women [101]. 47 studies included sex/gender as a factor in the regression analysis, but very few (3 studies [4.2%]) conducted advanced modeling techniques with the sex/gender variable. Ten studies (13.9%) referred to sex/gender-related research or theories when interpreting their findings. Among the topics addressed were gender roles [95] caretaking labors [41, 95, 97] work-family conflicts [53, 76, 103, 110] and gender stereotypes of masculinity and femininity [74, 110]. One article highlighted the importance of introducing a gender perspective for the mental health of both men and women [87], even without clearly including a gender theoretical framework in the discussion.

**Fig. 3:**
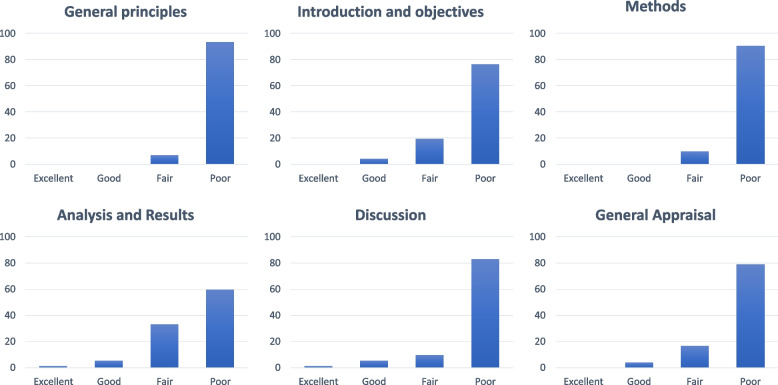
Sections of the appraisal tool and General appraisal. Each graphic shows the proportion of papers rated as poor, fair, good, and excelent

Of note, we identified gender stereotypes in three studies (4.2%). Beneria et al. [39] explain that “women had more symptoms of stress, probably related to the […] frustration with the death of patients whom they care”. The second example is that of Simonetti et al. [71] when they state that “higher levels of anxiety in female nurses are due to worries about infecting their children” and continue with “Higher self-efficacy in males probably [due to] their ability to solve problems and find solutions". In the third identified study [92], they note that “women [are] biologically more disposed to develop higher levels of anxiety and PTSD than men”, with no reference to social aspects. Finally, regarding the overall assessment of gender sensitivity, we rated only three papers (4.2%) as good [30, 74, 97]. *N* = 12 articles were rated as fair (16,7%), and the remaining as poor (*n* = 57 studies, [79,2%]). We did not identify any papers with excellent gender sensitivity (Fig. 3). There weren’t statistical differences in the proportion of women in the first or second author respecting gender sensitivity (*p* > 0,05).

## Discussion

To the best of our knowledge, this is the first attempt to comprehensively assess the gender sensitivity of COVID-19 research on the mental health of hospital staff. Our results suggest that, in general, gender sensitivity is low. Of the 72 studies included in the analysis, only three were rated as good in terms of gender sensitivity. Most of the studies suffered from important methodological flaws, such as using sex and gender erroneously or interchangeably, not specifying how the sex/gender of participants was determined, and lacking sex-disaggregated data. In most cases, non-binary individuals were not considered, nor were other variables such as migrant background or sexual orientation, which help identify marginalized identities within and between genders. In the discussion, very few articles used theoretical frameworks to situate their findings in relation to the gendered psychological impact of the pandemic and, in three articles, the results were explained based on gender stereotypes.

In our review, we assessed the rigor with which the concepts of sex and gender are treated. Although the vast majority of the articles reported the variables of sex or gender in their study, the reality is that more than 80% misused these concepts. This confusion is a widespread phenomenon that occurs even in gender-specific medical journals, where one would expect greater precision [111, 112]. For this reason, various institutions, including the EU, have been committed to promoting the correct distinction between the two [22, 23, 36, 113]. Another important finding is the lack of non-binary options for reporting the sex or gender variable. Only one study offered the option of "diverse" [107] and three “Other/prefer not to say” [46, 54, 66], terms that not clearly reflect the existing variability [114]. One claimed to have eliminated from the analysis individuals who did not identify as either man or woman [105] without specifying how these individuals were identified. In general, non-binary individuals are known to suffer higher rates of suicide, depression, and anxiety disorders [114] and in the case of healthcare workers, they are victims of discrimination and unable to disclose their identity [115, 116]. The fact that both the identification and analysis of trans or non-binary individuals has been neglected reflects the need for greater visibility of this population.

We only identified 14 studies with adequate sex/gender proportions [30, 43, 50, 54, 57, 60, 66, 74, 75, 83, 84, 94, 100, 107]. Like other authors [117], we considered that an article had an adequate proportion of men and women in two cases: in the case of proportions between 40 and 60%, or if the authors justified the reason for the sample having unequal values. During the pandemic more than 70% of health care workers were women [118], so it is possible that in many studies the proportions corresponded to those of the study population. However, the investigators should have clarified the reasons why the proportions were not equal in their sample, for example, by referring to the study population. As other authors have argued, the selection of subjects should seek the best number to facilitate the validity and representativeness of the results, even at the cost of unequal gender proportions [119], and this was not the case in almost 80% of the included studies. Methodological decisions on sex and gender in relation to the study population should be reported and justified [33, 120], as failure to do so may lead to unrepresentative results. For example, in clinical trials of acute coronary syndrome, the overrepresentation of men/males led to extrapolation of incorrect conclusions in women [121].

The way sex and gender were reported and included in the methodology was also assessed. Sex/gender disaggregated data is one-way researchers can discover differences in outcome measurements. In addition, it is one of the steps recognized by the EU Commission for the development of gender statistics. We found that only 37.5% of the included studies disaggregate data by sex or gender [30, 38, 41, 48, 53, 58, 59, 64, 68–71, 75, 74, 77, 82, 83, 88, 92, 95–99, 102, 104, 105]. This percentage is, however, higher than in clinical trials registered in COVID-19, where only 17.8% of the registered studies disaggregated the data [122] as well as in clinical trials published in high-impact journals, where the proportion drops to 14.0% [117]. In a study on authorship and sex disaggregation of data in COVID-19 research published in Spanish journals, the proportion was as low as 1% [123]. The reasons for these differences remain unknown. It is important for future research to determine whether EU policies had a positive influence on mental health research conducted during the pandemic. We then assessed what percentage of articles performed advanced statistical analysis with the variable of sex or gender. The underlying theory is that controlling for sex or gender treats these variables as confounders, rather than variables of importance to the research question. Ostensibly, it allows sex or gender differences in the outcome to be assessed, but it also forces this difference to be the same at all levels of the predictor [124]. This was, in fact, the most common way to include sex/gender in regression analysis. In our opinion, a more advanced approach would be to assess whether sex or gender is moderated or intersects with other variables [125], models that we only identified in three articles. Additionally, three other studies reported ethnicity and migration [107], but none subsequently performed any intersectional analysis.

One of our main findings is the identification of gender stereotypes in peer-reviewed publications. Gender stereotypes are general expectations and overgeneralized beliefs about people's characteristics based solely on their sex [126, 127]. In one of the studies, for example, the authors claim that women were more stressed by worrying about deceased patients, without evidence to substantiate this claim [39]. The perpetuation of the stereotype of masculinity as cold and emotionless precludes further development of programs for the male population. Indeed, they too were undoubtedly affected by patient deaths during the pandemic, but were less likely to seek support given the traditional male norm of being strong, in control, and able to avoid emotions [128]. In another example the authors state that the higher levels of anxiety found in female nurses were due to concern about infecting their children [71]. They go on to report that men had higher self-efficacy scores due to their ability to solve problems and find solutions. This statement reflects the primary importance we place on task performance when judging men and on social relationships when considering women [127]. Moreover, they also reinforce the gendered expectation that children are women's (and not men's) priority. In the last example, the authors attribute higher anxiety and PTSD in female health care workers to inherent biological factors [92] However, the authors do not mention the social factors that influence the poorer mental health of women in the healthcare sector. For example, problems in reconciling work and family life have been related to higher depression symptoms in women doctors [129]. In addition, they are victims of significant levels of workplace harassment and violence [130, 131], which predisposes them to a higher risk of developing PTSD symptoms when exposed to new traumatic experiences, such as the pandemic.

In our review most studies focused on internalizing disorders. Externalizing behaviors, such as drug use, have been little studied, or even undetected, in this population. Given that men are more likely to engage in risky behaviors in stressful situations [132], the impact of the pandemic on male healthcare workers may be underestimated. In addition, the fact that research focuses primarily on internalizing disorders may mask a stereotypical idea of femininity illness in women-dominated field such as medicine. Women also engage in substance abuse behavior, but it is stigmatized behavior and tends to be hidden [133]. In addition, men are less likely to seek psychiatric care and disclose mental health symptoms [134, 135], but the influence of masculinity on symptom reporting was only superficially mentioned in one article [74]. Present research will determine mental health interventions for healthcare workers in future pandemics. If knowledge production is biased, it may produce inaccurate results and the subsequent mental health programs may not be effective.

Our study has, however, some limitations. The inclusion of EU studies facilitates contextualization of the findings but may affect their generalizability. There are more institutions at the international level that also promote the inclusion of the gender dimension. Examples are the Canadian Institute of Health Research [136] or the U.S. National Institute of Health [113]. Other regions, on the contrary, do not have public policies aimed at integrating gender in research. Since there is a wide variation by country, conclusions should be drawn with caution. A second limitation is the focus on hospital staff. It is possible that gender sensitivity in studies of mental health in outpatient staff or in the general population may be different. In addition, tools for assessing the integration of sex/gender in research studies need to be further developed in the future.

## Conclusions

Our study shows that most European research on the psychological impact of COVID-19 on hospital staff is insufficiently sensitive to sex/gender, even after a clear public commitment by the EU. Gender biases may be present from study design to interpretation of results, and this may interfere with the development of effective prevention and treatment interventions in future pandemics. The impact on non-binary individuals was neglected and remains unknown, as is the interplay between gender and other variables such as occupation, ethnicity, or sexual orientation since no interaction analyses were performed. Our findings call for a greater inclusion of the sex/gender dimension in future research to develop effective interventions in future pandemics.

## Supplementary Information

Supplementary Material 1.

## Data Availability

This work analyzed secondary sources, which are cited in the manuscript. Additional data from the analysis can be requested to the corresponding author.
