# Supplementary material for: Gender sensitivity of the COVID-19 mental health research in Europe: a scoping review
Source: Int J Equity Health. 2024 Oct 10;23:207. doi: 10.1186/s12939-024-02286-1 (PMC11465889; doi:10.1186/s12939-024-02286-1)
Supplement: Supplementary file 1 — Supplementary Material 1. [file 12939_2024_2286_MOESM1_ESM.docx]

[Apendix 1: Study protocol 2](#_Toc139275708)

[General information 3](#_Toc139275709)

[1. Title 3](#_Toc139275710)

[2. Research team and organizational affiliations 3](#_Toc139275711)

[3. Timeline 3](#_Toc139275712)

[4. Funding sources 3](#_Toc139275713)

[Project details according to the PRISMA-ScR checklist 4](#_Toc139275714)

[Item 1. Title 4](#_Toc139275715)

[Item 2. Abstract 4](#_Toc139275716)

[Item 3. Rationale 4](#_Toc139275717)

[Item 4. Objectives 5](#_Toc139275718)

[Item 5. Protocol and registration 5](#_Toc139275719)

[Item 6. Eligibility criteria 5](#_Toc139275720)

[Item 7. Information sources 6](#_Toc139275721)

[Item 8. Search 6](#_Toc139275722)

[Item 9. Selection of sources of evidence 6](#_Toc139275723)

[Item 10. Data charting process 7](#_Toc139275724)

[Item 11. Data items 7](#_Toc139275725)

[Item 12. Critical appraisal of individual sources of evidence 7](#_Toc139275726)

[Item 13. Synthesis of results: 8](#_Toc139275727)

[Items 14-21: Results / Discussion 8](#_Toc139275728)

[Item 22: Funding 8](#_Toc139275729)

[PRISMA extension for scoping reviews (PRISMA-ScR) checklist 8](#_Toc139275730)

[Apendix2: Search strategy 11](#_Toc139275731)

[Apendix 3: Sex/gender appraisal tool 13](#_Toc139275732)

[Apendix 4: Inter rater analysis: 21](#_Toc139275733)

[Apendix 5: List of included articles 22](#_Toc139275734)

[Apendix 6: List of excluded articles: 25](#_Toc139275735)

[Bibliography: 31](#_Toc139275736)

### Apendix 1: Study protocol

**Table of contents**

[Apendix 1: Study protocol 2](#_Toc140130475)

[General information 4](#_Toc140130476)

[1. Title 4](#_Toc140130477)

[2. Research team and organizational affiliations 4](#_Toc140130478)

[3. Timeline 4](#_Toc140130479)

[4. Funding sources 4](#_Toc140130480)

[Project details according to the PRISMA-ScR checklist 5](#_Toc140130481)

[Item 1. Title 5](#_Toc140130482)

[Item 2. Abstract 5](#_Toc140130483)

[Item 3. Rationale 5](#_Toc140130484)

[Item 4. Objectives 6](#_Toc140130485)

[Item 5. Protocol and registration 6](#_Toc140130486)

[Item 6. Eligibility criteria 6](#_Toc140130487)

[Item 7. Information sources 7](#_Toc140130488)

[Item 8. Search 7](#_Toc140130489)

[Item 9. Selection of sources of evidence 7](#_Toc140130490)

[Item 10. Data charting process 8](#_Toc140130491)

[Item 11. Data items 8](#_Toc140130492)

[Item 12. Critical appraisal of individual sources of evidence 8](#_Toc140130493)

[Item 13. Synthesis of results: 9](#_Toc140130494)

[Items 14-21: Results / Discussion 9](#_Toc140130495)

[Item 22: Funding 9](#_Toc140130496)

[PRISMA extension for scoping reviews (PRISMA-ScR) checklist 9](#_Toc140130497)

[Apendix2: Search strategy 11](#_Toc140130498)

[Apendix 3: Sex/gender appraisal tool 13](#_Toc140130499)

[Apendix 4: Inter rater analysis: 21](#_Toc140130500)

[Apendix 5: List of included articles 22](#_Toc140130501)

[Apendix 6: List of excluded articles: 25](#_Toc140130502)

[Bibliography: 31](#_Toc140130503)

# General information

## 1. Title

Gender sensitivity in studies of healthcare workers’ mental health during the COVID-19 pandemic: a scoping review.

## 2. Research team and organizational affiliations

1. Dr. Margarita Sáenz Herrero^1,2^
2. Mayte López Atanes^1,2^
3. Dr. Tilman Brand^3^
4. Elisa Fraile García^1^
5. Dr. Rafael Segarra Echevarria^1,2^
6. Lara Christianson^3^

^1^Department of Psychiatry. Cruces University Hospital. Osakidetza-Basque Health Service, Barakaldo (Spain).

^2^Department of Neurosciences. School of Medicine and Nursing. University of the Basque Country (UPV/EHU), Leioa (Spain).

^3^ Leibniz Institute for Prevention Research and Epidemiology – BIPS, Department Prevention and Evaluation. Bremen (Germany).

## 3. Timeline

1. Start date: 1.12.2021
2. Anticipate completion date: 1.12.2022

## 4. Funding sources

No funding.

# Project details according to the PRISMA-ScR checklist

## Item 1. Title

Gender sensitivity in studies of healthcare workers’ mental health during the COVID-19 pandemic: a scoping review.

## Item 2. Abstract

Not applicable at the protocol stage.

## Item 3. Rationale

The coronavirus pandemic was a distressing event for healthcare workers. Depression, anxiety, and suicidal ideation were common among the medical population, especially women in lower-paid positions and those working in the frontline against the virus. According to the WHO, women form 70% of the medical population, and due to occupational segregation, they tend to engage in lower-paid positions such as nursing or nursing assistance. For this reason, women were more likely to be occupationally exposed to the SARS-COV-19 virus.

Psychosocial variables can contribute to the poorer mental health of women in the healthcare sector. They are subject to gender-related stressors that became more pronounced during the pandemic. The number of abuse and domestic violence cases increased and also caretaking labours, which are delegated mainly to women due to the closing of schools and day-care centers. Women in the frontline against the virus, in particular, also had to deal with excessive stress at work and the risk of infection.

Most current studies of the psychological impact of the pandemic fail to incorporate gender-sensitive methodology into research. In research, the lack of gender sensitivity or androcentrism may cause systematic gender bias during the design and analysis of the data. It may occur at any stage of the inferential process. If we focus on the medical population, published work barely alludes to gender, fails to provide gender-disaggregated data, and does not include gender-related variables in the discussion to interpret the results. We want to analyze whether there is sex/gender bias in the studies of the psychological impact of the pandemic on healthcare workers and check whether a gender-sensitive analysis drives to different conclusions or a better interpretation of the data compared to gender-blind research.

## Item 4. Objectives

The following research questions were formulated:

- Is current literature about the psychological impact of the coronavirus on healthcare workers gender-sensitive?
- Are results and conclusions gender-biased?
- Does gender-sensitive research have different outcomes or conclusions?

## Item 5. Protocol and registration

We drafted our protocol following the Preferred Reporting Items for Systematic reviews and meta-Analysis extension for Scoping Reviews (PRISMA-ScR) Checklist (Tricco et al., 2018; refer to the Appendix). The protocol will be prospectively registered at the Center for Open Science (OSF): <https://osf.io/>.

## Item 6. Eligibility criteria

The eligibility criteria for this scoping review are based on the PCC (Population, Concept and Context) criteria recommended for scoping reviews:

**Inclusion criteria:**

1. Study designs: Primary quantitative studies. It would be expected to be observational rather than experimental studies.
2. Publication status: published in peer-reviewed journals or other sources (e.g., organizational reports) starting on the 1st of December 2019 to the date of the search.
3. ***P: P****opulation*: Hospital-based healthcare workers in direct or indirect contact with the SARS-COV-19 virus.
4. ***C: C****oncept*: The primary outcome is evaluating the mental health or psychological distress of healthcare workers during the COVID-19 pandemic. It can be assessed using depression, anxiety, stress, PTSD, quality of life, or suicidal ideation validated scales. A validated scale refers to a questionnaire/scale that has been developed to be administered among the intended respondents. The validation process should demonstrate adequate reliability and and validity.

As secondary outcomes, we will include studies that address other aspects related to the coronavirus infection or scales not yet validated.

1. ***C: C****ontext*: Hospital-based studies.

**Exclusion criteria:**

1. No primary data: literature reviews, letters, editorials, comments, book reviews, monographs.
2. Qualitative studies.
3. Studies that include healthcare workers in ambulatory settings.
4. Other target populations such as students (including medical students) or the general population.
5. Conference abstracts.
6. Other languages than English, German or Spanish.
7. Full-text not accessible.

## Item 7. Information sources

The following bibliographic databases will be searched from inception to January 2022:

1. MEDLINE via OvidSP.
2. EMBASE via OvidSP
3. CINAHL via EBSCO.
4. PsycInfo via OvidSP
5. Science citation index expanded via Web of Science.
6. Social Sciences Citation Index (SSCI) via Web of Science.

## Item 8. Search

The search will be conducted in English. No language or date limitations will be set at the search stage. We aim to include sources in English, German or Spanish. The search strategy will be developed iteratively by the team that includes a professional librarian. The search terms will reflect the PCC criteria relevant for this scoping review:

**Population** (healthcare workers) AND **concept** (mental health) AND **context** (hospital-based studies)

## Item 9. Selection of sources of evidence

The professional librarian will search the listed databases on our team, and the search results will be saved in the reference management software, Endnote. After deduplication in Endnote, the remaining results will be exported to the literature review software Rayyan. Following a further check for duplicates in Rayyan, the scoping review will be done in two stages: (i) screening of titles and abstracts and (ii) screening of full texts of the studies included in the next stage. Two reviewers will do both stages independently based on the inclusion and exclusion criteria set for this scoping review. Any discrepancies will be resolved during discussion until consensus is reached.

## Item 10. Data charting process

We will develop a data-charting form for this scoping review. The team will discuss and agree upon how data items will be selected, and the software used, and will test and refine the charting form to ensure that all relevant data will be captured. The complete data charting will be conducted independently by two team members. Any discrepancies will be discussed until consensus is reached.

## Item 11. Data items

The following data items will be abstracted:

- Generic bibliographic information: (e.g., country of origin, funder, date of publication)
- Sex/Gender of the first and last authors, if known.
- Design and study aims.
- Characteristics of the study population.
- Measurement of the psychological impact of the pandemic or mental health. Measures of sex/gender sensitivity: (e.g., Sex/Gender disaggregation of data, the inclusion of gender-related variables in the analysis).

## Item 12. Critical appraisal of individual sources of evidence

We will appraise the quality of cross-sectional studies using the NHI Quality Assessment Tool for Observational Cohort and Cross-Sectional Studies. In addition, experimental studies will be assessed with the NHI Quality Assessment Tool of Controlled Intervention Studies. Furthermore, we will develop a sex/gender appraisal tool based on the Epi goes gender Project evaluation sheet(1) to check the degree of gender sensitivity of the included studies.

## Item 13. Synthesis of results:

A summary of study characteristics and results will be provided. We will summarize the gender sensitivity of the studies according to a chart developed by the team. Recommendations for best-practice procedures will be formulated.

## Items 14-21: Results / Discussion

**Not applicable at the protocol stage.**

## Item 22: Funding

This project received no funding

## PRISMA extension for scoping reviews (PRISMA-ScR) checklist

Tricco AC, Lillie E, Zarin W, O'Brien KK, Colquhoun H, Levac D, et al. PRISMA extension for scoping reviews (PRISMA-ScR): checklist and explanation. Annals of Internal Medicine. 2018;169(7):467-73. doi:[10.7326/M18-0850](https://doi.org/10.7326/M18-0850)

| **Section** | **Item** | **PRISMA-ScR Checklist Item** | **Page** |
| --- | --- | --- | --- |
| Title | 1 | Identify the report as a scoping review. | 5 |
| **Abstract** |  |  |  |
| Structured summary | 2 | Provide a structured summary that includes (as applicable): background, objectives, eligibility criteria, sources of evidence, charting methods, results, and conclusions that relate to the review questions and objectives. | 5 |
| **Introduction** |  |  |  |
| Rationale | 3 | Describe the rationale for the review in the context of what is already known. Explain why the review questions/objectives lend themselves to a scoping review approach. | 5 |
| Objectives | 4 | Provide an explicit statement of the questions and objectives being addressed with reference to their key elements (e.g., population or participants, concepts, and context) or other relevant key elements used to conceptualize the review questions and/or objectives. | 6 |
| **Methods** |  |  |  |
| Protocol and registration | 5 | Indicate whether a review protocol exists; state if and where it can be accessed (e.g., a Web address); and if available, provide registration information, including the registration number. | 6 |
| Eligibility criteria | 6 | Specify characteristics of the sources of evidence used as eligibility criteria (e.g., years considered, language, and publication status), and provide a rationale. | 6 |
| Information sources | 7 | Describe all information sources in the search (e.g., databases with dates of coverage and contact with authors to identify additional sources), as well as the date the most recent search was executed. | 7 |
| Search | 8 | Present the full electronic search strategy for at least 1 database, including any limits used, such that it could be repeated. | 7 |
| Selection of sources of evidence | 9 | State the process for selecting sources of evidence (i.e., screening and eligibility) included in the scoping review. | 7 |
| Data charting process | 10 | Describe the methods of charting data from the included sources of evidence (e.g., calibrated forms or forms that have been tested by the team before their use, and whether data charting was done independently or in duplicate) and any processes for obtaining and confirming data from investigators. | 8 |
| Data items | 11 | List and define all variables for which data were sought and any assumptions and simplifications made. | 8 |
| Critical appraisal of individual sources of evidence | 12 | If done, provide a rationale for conducting a critical appraisal of included sources of evidence; describe the methods used and how this information was used in any data synthesis (if appropriate). | 8 |
| Synthesis of results | 13 | Describe the methods of handling and summarizing the data that were charted. | 9 |
| **Results** |  |  |  |
| Selection of sources of evidence | 14 | Give numbers of sources of evidence screened, assessed for eligibility, and included in the review, with reasons for exclusions at each stage, ideally using a flow diagram. | 9 |
| Characteristics of sources of evidence | 15 | For each source of evidence, present characteristics for which data were charted and provide the citations. | 9 |
| Critical appraisal within sources of evidence | 16 | If done, present data on critical appraisal of included sources of evidence (see item 12). | 9 |
| Results of individual sources of evidence | 17 | For each included source of evidence, present the relevant data that were charted that relate to the review questions and objectives. | 9 |
| Synthesis of results | 18 | Summarize and/or present the charting results as they relate to the review questions and objectives. | 9 |
| **Discussion** |  |  |  |
| Summary of evidence | 19 | Summarize the main results (including an overview of concepts, themes, and types of evidence available), link to the review questions and objectives, and consider the relevance to key groups. | 9 |
| Limitations | 20 | Discuss the limitations of the scoping review process. | 9 |
| Conclusions | 21 | Provide a general interpretation of the results with respect to the review questions and objectives, as well as potential implications and/or next steps. | 9 |
| **Funding** |  |  |  |
| **Funding** | 22 | Describe sources of funding for the included sources of evidence, as well as sources of funding for the scoping review. Describe the role of the funders of the scoping review. | 9 |

1. Jahn I, Börnhorst C, Günther F, Brand T. Examples of sex/gender sensitivity in epidemiological research: results of an evaluation of original articles published in JECH 2006–2014. Heal Res Policy Syst [Internet]. 2017;15(1):11. Available from: https://doi.org/10.1186/s12961-017-0174-z

### Apendix2: Search strategy

| **MEDLINE via ovid** |
| --- |
| 1 (coronavirus or "COVID-19" or "SARS-CoV-2" or "COVID").ti,ab. 198815 |
| 2 exp COVID-19/ 117738 |
| 3 exp SARS-CoV-2/ 92295 |
| 4 (depress* or anxiety or stress* or distress* or "post-traumatic stress disorder*" or "PTSD" or "mental health" or "substance abuse" or "risk behaviour" or "risk behavior" or "alcohol abuse" or insomnia or suicid*).ti,ab. 1762450 |
| 5 exp Mental disorders/ 1331759 |
| 6 (doctor* or nurs* or "healthcare worker*" or "medical staff" or physician* or "nursing assistant*" or "healthcare professional*" or "medical workforce" or "hospital worker*" or "hospital staff" or "health professional*" or "health provider*").ti,ab. 1058312 |
| 7 exp Health personnel/ 563462 |
| 8 (hospital* or "tertiary center*" or "tertiary centre*" or "secondary center*" or "secondary centre*" or "unit" or "tertiary care" or "secondary care" or frontline).ti,ab. 1790452 |
| 9 exp Hospitals/ 294965 |
| 10 exp Hospital units/ 123783 |
| 11 1 or 2 or 3 204763 |
| 12 4 or 5 2734660 |
| 13 6 or 7 1380006 |
| 14 8 or 9 or 10 1940399 |
| 15 11 and 12 and 13 and 14 1858 |
|  |
| **EMBASE via ovid** |
| 1 (coronavirus or "COVID-19" or "SARS-CoV-2" or "COVID").ti,ab. 200927 |
| 2 exp coronavirus disease 2019/ 159905 |
| 3 (depress* or anxiety or stress* or distress* or "post-traumatic stress disorder*" or "mental health" or "substance abuse" or "risk behaviour" or "risk behavior" or "alcohol abuse" or insomnia or suicid*).ti,ab. 2256205 |
| 4 exp mental disease/ 2356911 |
| 5 (doctor* or nurs* or "healthcare worker*" or "medical staff" or physician* or "nursing assistant*" or "healthcare professional*" or "medical workforce" or "hospital worker*" or "hospital staff").ti,ab. 1287666 |
| 6 exp Health care personnel/ 1719211 |
| 7 (hospital* or "tertiary center*" or "tertiary centre*" or "secondary center*" or "secondary centre*" or "unit" or "tertiary care" or "secondary care" or frontline).ti,ab. 2660652 |
| 8 exp Hospital/ 1238961 |
| 9 exp Tertiary care center/ 78476 |
| 10 exp secondary care center/ 1772 |
| 11 1 or 2 215292 |
| 12 3 or 4 3858248 |
| 13 5 or 6 2411290 |
| 14 7 or 8 or 9 or 10 3133702 |
| 15 11 and 12 and 13 and 14 3461 |
| 16 limit 15 to exclude medline journals 738 |
|  |
| **Psyinfo via ovid** |
| 1 (coronavirus or "COVID-19" or "SARS-CoV-2" or "COVID").ti,ab. 10996 |
| 2 exp coronavirus/ 3549 |
| 3 (depress* or anxiety or stress* or distress* or "post-traumatic stress disorder*" or "PTSD" or "mental health" or "substance abuse" or "risk behaviour" or "risk behavior" or "alcohol abuse" or insomnia or suicid*).ti,ab. 892394 |
| 4 exp Mental disorders/ 908342 |
| 5 (doctor* or nurs* or "healthcare worker*" or "medical staff" or physician* or "nursing assistant*" or "healthcare professional*" or "medical workforce" or "hospital worker*" or "hospital staff" or "health professional*" or "health provider*").ti,ab. 235756 |
| 6 exp Health personnel/ 173691 |
| 7 (hospital* or "tertiary center*" or "tertiary centre*" or "secondary center*" or "secondary centre*" or "unit" or "tertiary care" or "secondary care" or frontline).ti,ab. 214689 |
| 8 exp Hospitals/ 25732 |
| 9 1 or 2 11305 |
| 10 3 or 4 1435366 |
| 11 5 or 6 330212 |
| 12 7 or 8 218040 |
| 13 9 and 10 and 11 and 12 367 |
|  |
| **Web of science:** |
| 1. (TI=(coronavirus or “COVID-19” or “SARS-CoV-2” or “COVID”)) OR AB=(coronavirus or “COVID-19” or “SARS-CoV-2” or “COVID”) |
| 1. (TI=((depress* or anxiety or stress* or distress* or "post-traumatic stress disorder*" or "PTSD" or "mental health" or "substance abuse" or "risk behaviour" or "risk behavior" or "alcohol abuse" or insomnia or suicid*))) OR AB=((depress* or anxiety or stress* or distress* or "post-traumatic stress disorder*" or "PTSD" or "mental health" or "substance abuse" or "risk behaviour" or "risk behavior" or "alcohol abuse" or insomnia or suicid*)) |
| 1. (TI=((doctor* or nurs* or "healthcare worker*" or "medical staff" or physician* or "nursing assistant*" or "healthcare professional*" or "medical workforce" or "hospital worker*" or "hospital staff" or "health professional*" or "health provider*"))) OR AB=((doctor* or nurs* or "healthcare worker*" or "medical staff" or physician* or "nursing assistant*" or "healthcare professional*" or "medical workforce" or "hospital worker*" or "hospital staff" or "health professional*" or "health provider*")) |
| 1. (TI=(hospital* or "tertiary center*" or “tertiary centre*” or "secondary center*" or “secondary centre*” or "unit" or "tertiary care" or "secondary care" or frontline)) OR AB=(hospital* or "tertiary center*" or “tertiary centre” or “secondary centre” or "secondary center*" or "unit" or "tertiary care" or "secondary care" or frontline) |
| 1. (((#1) AND #2) AND #3) AND #4 |
| **Results: 1178** |
|  |
| **CINAHL** |
| - TI ( coronavirus or "COVID-19" or "SARS-CoV-2" or "COVID" ) OR AB ( coronavirus or "COVID-19" or "SARS-CoV-2" or "COVID" ) |
| - **(MH "COVID-19") OR (MH "SARS-CoV-2")** |
| - TI ( depress* or anxiety or stress* or distress* or "post-traumatic stress disorder*" or "PTSD" or "mental health" or "substance abuse" or "risk behaviour" or "risk behavior" or "alcohol abuse" or insomnia or suicid* ) OR AB ( depress* or anxiety or stress* or distress* or "post-traumatic stress disorder*" or "PTSD" or "mental health" or "substance abuse" or "risk behaviour" or "risk behavior" or "alcohol abuse" or insomnia or suicid* ) |
| - **(MH "Behavioral and Mental Disorders+")** |
| - TI ( doctor* or nurs* or "healthcare worker*" or "medical staff" or physician* or "nursing assistant*" or "healthcare professional*" or "medical workforce" or "hospital worker*" or "hospital staff" or "health professional*" or "health provider*" ) OR AB ( doctor* or nurs* or "healthcare worker*" or "medical staff" or physician* or "nursing assistant*" or "healthcare professional*" or "medical workforce" or "hospital worker*" or "hospital staff" or "health professional*" or "health provider*" ) |
| - **(MH "Health Personnel+")** |
| - TI ( hospital* or "tertiary center*" or “tertiary centre*” or "secondary center*" or “secondary centre*” or "unit" or "tertiary care" or "secondary care" or frontline ) OR AB ( hospital* or "tertiary center*" or “tertiary centre*” or "secondary center*" or “secondary centre*” or "unit" or "tertiary care" or "secondary care" or frontline ) |
| - (MH "Hospitals+") |
| - (MH "Hospital Units+") |
| 917 results |

### Apendix 3: Sex/gender appraisal tool

| **GENERAL PRINCIPLES** | Yes | No | Other (Not applicable, unclear) |
| --- | --- | --- | --- |
| 1. Were sex or gender considered at all? | □ | □ | □ |
| 1. Were sex and gender defined? | □ | □ | □ |
| 1. **Does the paper mention gender categories besides the woman/man binary?** | □ | □ | □ |
| 1. Were the terms sex/gender correctly used in the article? | □ | □ | □ |
| METHODOLOGY | | | |
| **TITLE AND ABSTRACT** | Yes | No | Other (Not applicable, unclear) |
| 1. If only one sex/gender was included, was this made clear in the title/abstract? | □ | □ | □ |
| **INTRODUCTION AND OBJECTIVES** |  |  |  |
| 1. Does the literature review include sex/gender differences or similarities? | □ | □ | □ |
| 1. Were sex/gender mentioned in the objectives of the study? | □ | □ | □ |
| 1. Did the authors explain what aspects of sex/gender were analyzed? | □ | □ | □ |
| **METHODS** |  |  |  |
| 1. Were other sex/gender categories beyond the men/women binary included in the data collection? |  |  |  |
| 1. Are other gender-related variables captured in the study? | □ | □ | □ |
| 1. Did the authors consider the gender sensitivity of the tools used in the study? | □ | □ | □ |
| **RESULTS AND ANALYSIS** |  |  |  |
| 1. Is data presented disaggregated by sex/gender? | □ | □ | □ |
| 1. Is there an adequate representation of women and men? | □ | □ | □ |
| 1. Was sex/gender appropriately included as a factor a regression analys? | □ | □ | □ |
| 1. Were sex/gender differences analyzed using advanced modeling techniques (like interaction analysis)? | □ | □ | □ |
| **DISCUSSION** |  |  |  |
| 1. Were the findings reflected concerning sex/gender? | □ | □ | □ |
| 1. Were gender stereotypes present in the interpretation of the data? | □ | □ | □ |
| 1. Were there any inadequate generalizations in the study? | □ | □ | □ |

**Appraisal of each section and overall appraisal:**

|  | Excellent | Good | Fair | Poor |
| --- | --- | --- | --- | --- |
| GENERAL PRINCIPLES | □ | □ | □ | □ |
| INTRODUCTION AND OBJECTIVES | □ | □ | □ | □ |
| METHODS | □ | □ | □ | □ |
| RESULTS AND ANALYSIS | □ | □ | □ | □ |
| DISCUSSION AND CONCLUSIONS | □ | □ | □ | □ |
|  | Excellent | Good | Fair | Poor |
| **OVERALL APPRAISAL** | □ | □ | □ | □ |

**The purpose of this tool is to assess the gender sensitivity of an article. First, the evaluator must read one example of good gender sensitivity (1) and one example of poor gender sensitivity (2). Then, the evaluation is performed in three steps:**

1. Evaluation of general principles of sex and gender categorization.
2. Evaluation of the different steps of the research process by means of yes/no questions.
3. Overall qualitative evaluation of each section and final evaluation of the whole document.
4. Mele BS, Holroyd-Leduc JM, Harasym P*, et al* Healthcare workers’ perception of gender and work roles during the COVID-19 pandemic: a mixed-methods study BMJ Open 2021;11:e056434. doi: 10.1136/bmjopen-2021-056434
5. Lai J, Ma S, Wang Y, et al. Factors Associated With Mental Health Outcomes Among Health Care Workers Exposed to Coronavirus Disease 2019. *JAMA Netw Open.* 2020;3(3):e203976. doi:10.1001/jamanetworkopen.2020.3976

**Discussion of the items:**

***GENERAL PRINCIPLES:***

1. **Was sex or gender considered at all?**

*Rationale and examples:* Does the article consider sex or gender in any aspect? This question is intended to detect those articles that completely ignore sex or gender in their study. Examples of terms related to sex and gender are sex, gender, transgender, gender-fluid, non-binary, sex/gender, male/s, or female/s, man, men, woman, women, boy/s, girl/s.

- Yes: The document considers sex or gender at some point in the article.
- No: The paper does not consider sex or gender at any point.
- Other/Not applicable: The reviewer cannot answer the question or considers that it does not apply to the article.

1. **Was sex/gender defined?**

*Rationale and examples:* Sex refers to the biologically determined characteristics of males and females in terms of reproductive organs and functions, and it is based on chromosomal complement and physiology. Sex is understood globally as the classification of living beings into **male or female.** Conceptually it is based on the female/male binary, although in reality chromosomal variability is much broader (1). Sex can also be conceptualized in interaction with gender, rather than as a static difference between females and males. Gender refers to the social construction of femininity and masculinity, which varies over time and place and across cultures (2). As a static concept, it is defined as a person’s self-representation as women or men, but relational theory describes it as a multidimensional concept. (1) The key concepts are that sex is about biology and gender is about social constructs.

- **Yes**: the study provides **some** definition of sex/gender.
- **No**: the study does not provide **any** definition of sex and/or gender.
- **Other/Not applicable:** The evaluator cannot answer this question, it does not apply to the article or the definitions are not correct according to the evaluator (e.g., defining gender as the chromosomal type of the individual).

1. **Does the paper mention sex or gender categories other than the binary?**

*Rationale and examples:* This question asks whether the article mentions non-binary individuals in any way. In medical research, sex and gender are usually categorized as binary (women/men, girl/boy, male/female) but there are also non-binary identities that should be included in research (e.g., non-binary, intersex, trans). Gender identity describes how an individual sees themselves in terms of social roles, aspirations, behaviors, and body image, something that evolves and is not always stable.

- **Yes**: The article meets the criteria.
- **No**: The article does not meet the criteria.
- **Other:** The reviewer is unable to answer the question or considers that it does not apply to the article.

1. **Is sex/gender correctly used in the article?**

*Rationale and examples*: Does the study use the concepts of sex and gender appropriately? Sex/gender should be used according to the definition provided, if applicable. To describe the sex of a subject, the terms male or female should be used. Terms such as man, men, woman, women, boy/s, girl/s refer to the gender of the subject and should be used accordingly. Other denominations may be possible if justified.

- **Yes**: sex and gender are correctly used.
- **No**: sex or gender is not correctly used (e.g., used interchangeably or mistaking sex with gender or vice versa).
- **Other (unclear, not applicable):** The reviewer is unable to answer the question or considers that the question does not apply to the paper.

***TITLE AND ABSTRACT***

1. **If only one sex/gender was included, was it made clear in the title/abstract?**

*Rationale and examples:* If only one sex or gender is included in the study, the title and the abstract should specify this. For example, stating “in women/females“ or „in men/males“.

- **Yes**: the article meets the criteria.
- **No**: the article did not meet the criteria.
- **Other (unclear, not applicable):** The reviewer is unable to answer the question or considers that this question does not apply to the paper.

***INTRODUCTION AND OBJECTIVES***

1. **Does the literature review include sex/gender differences or similarities?**

*Rationale and examples:* Authors should report the presence or absence of studies assesing gender differences or similarities in the outcome measure, and also identify knowledge gaps if any. For example, in mental health, women tend to have more internalizing disorders, such as depression or anxiety, while men tend to present externalizing disorders such as alcohol abuse. If no sex/gender differences were found, it should also be specified. Are there any knowledge gaps or questions about sex or gender raised in the literature review?

- **Yes:** the authors review the evidence on sex/gender differences or similarities OR knowledge gaps, whether they exist or not.
- **No:** there is no mention of sex or gender at all.
- **Other (unclear, not applicable):** The reviewer annot answer the question or considers that the question does not apply to the paper.

1. **Was sex/gender mentioned in the objectives of the study?**

*Rationale and examples:* Are questions about sex/gender formulated in the research question? Are words such as sex, gender, male or female used? Related words would be sex, gender, male/s, female/s, man, men, woman, women, boy/s, girls/s.

- **Yes:** authors explicitly formulate questions about sex/gender in their objectives.
- **No:** authors do not formulate any question about sex/gender in their objectives.
- **Other: e.g** The reviewer is unable to determine the question.

1. **Did the authors explain what aspects of sex or gender are analyzed?**

Gender can be conceptualized from different aspects such as gender-related behaviors, roles, stereotypes, socialization, or discrimination. When exploring gender, authors should explain and justify which aspects they are analyzing.

- **Yes:** Authors explain the aspects of gender they analyze.
- **No:** Authors do not explain which aspects of gender they analyze.
- **Other/not applicable:** The reviewer is unable to answer this question or considers that it is not applicable to the paper (for example, if gender is not approached/categorized in the article.

***METHODS***

1. **Was the variable of sex/gender categorized beyond a dichotomy?**

*Rationale and examples:* The dichotomy of male/female, women/men do not capture the full spectrum of possibilities, such as non-binary individuals or transgender. Also, there is a risk of essentialism, that is, the tendency to generalize differences to all groups of people. When collecting data to describe the study sample, options beyond the dichotomy of male/female or women/men should be included.

- **Yes:** Sex/gender was formulated beyond a binary category. (options such as “prefer not to tell” should not be considered as a yes).
- **No:** sex/gender were formulated as a dichotomous variable.
- **Other (unclear, not applicable):** The reviewer is unable to answer the question or considers that it does not apply to the paper.

1. **Are gender-related variables captured in the study?**

*Rationale and examples:* A gender-related variable is a non-biological variable that differs in terms of magnitude, prevalence, and/or impact between people of a different gender. There are gender differences associated with ethnicity, religion, disability, and sexual orientation, as well as with migration, and citizenship status.

Authors should establish a list of gender-related variables relevant to their study and define relevant outcomes. To determine whether a variable is gender-related, it should be analyzed by introducing gender within the framework of the study. For example, when studying the psychological distress experienced by women in healthcare, including the number of hours dedicated to housing care in the study would work as a gender-related variable, as it has been previously described that women in the healthcare sector experience work-life balance difficulties that increase their depressive symptoms. In a study of drug adherence, income can work as a gender-related variable, as there are well-described gender differences in income that can affect the ability to pay for the drug.

- **Ye**s: the article meets the criteria.
- **No:** the article does not meet the criteria.
- **Other (unclear, not applicable):** The reviewer is unable to answer the question or considers that it does not apply to the paper (e.g. the authors provide an explanation for not including gender-related variables).

1. **Do authors consider the gender sensitivity of the tools used in the study?**

*Rationale and examples:* Psychometric tools can be subject to gender bias. This can be present in the construction of the instrument, the language used, the selection of the population or the toll, or in the evaluation itself. For example, psychometric scales addressing eating disorders usually address symptoms present in women than men are much less likely to present, such as a desire for thinness.

- **Yes**: the authors state that they evaluated the gender sensitivity of the tools used.
- **No:** the article did not meet the criteria.
- **Other (unclear, not applicable): T**he reviewer is unable to answer the question or considers that does not apply to the paper***.***

***RESULTS AND ANALYSIS***

1. **Is the data presented disaggregated by sex/gender?**

*Rationale and examples:* The key outcome of the study should be presented disaggregated by sex/gender. When results for male and female participants are combined, the average of aggregated male and female participants’ results may mask differences between them (4). For example, on an anxiety scale, the average score should be reported for women and men separately. If the score is the total average, that would mean the data is not disaggregated.

- **Yes:** the study met the criteria (outcome variable disaggregated respecting sex or gender).
- **No**: the study did not meet the criteria.
- **Other (unclear, not applicable):** The reviewer is unable to determine the question.

1. **Is there an adequate representation of women and men?**

*Rationale and examples:* According to the EU, an adequate proportion of women and men would be within 60-40% of the total sample (2). For example, 55% men and 45% would fit into this range, while 20% men and 80% would not. If the representation is justified based on the underlying distribution in a particular group, it should be considered adequate. For example, 70% of healthcare workers are women, according to the WHO, so a proportion similar to that would be adequate for respecting this particular group of people.

- **Yes:** There is an adequate proportion of women and men, OR the different proportions are justified by the distribution in the target population.
- **No:** The article did not meet the criteria.
- **Other: e.g.** The reviewer is unable to determine the question.

1. **Was sex/gender appropriately included as a factor in the statistical analysis?**

*Rationale and examples:* If sex/gender is included in the regression analysis to adjust for differences in the sex/gender distribution in the exposed (e.g. frontline staff) and unexposed group (other staff), the use is appropriate. If sex/gender is analyzed as the exposure/ variable of interest without considering differences in mental health outcomes that are already known to exist in the general population, then it is not appropriate because this can result in misleading interpretations. Thus, evaluating the appropriateness of the inclusion of sex/gender as a factor is not primarily a technical question, but a question of whether the inclusion serves the purpose (and interpretation) of what the researchers want to demonstrate.

- **Yes**: Sex/Gender is appropriately used as a factor (usually in regression analysis)
- **No**: gender is not used as a factor in the regression analysis.
- **Other/not applicable:** In this case, the authors explain why they do not include gender in the regression analysis or why they did not perform a regression analysis.

1. **Were sex/gender differences or similarities analyzed using advanced modeling techniques (like intersectional gender analysis)?**

*Rationale and examples:* In regression analysis, a variable may act as a moderator; that means, a determining variable has a different effect on the dependent variable depending on other covariates. This is analyzed through interaction regression analysis. Intersectional gender analysis represents how gender intersects with other characteristics within the intersectionality wheel, emphasizing gender as our main entry point into this analysis.

For example, if a study checks the different effects of the variable sex/gender respecting “tobacco use” and the dependent variable “cholesterol levels”, this study will be analyzing the differential effect that tobacco use has with respect to gender.

- **Yes**: the study provides gender intersectional analysis or includes gender as a moderator in the regression analysis.
- **No:** the intersectional analysis was not performed or sex/gender was not included in it.
- **Other/Not applicable:** Authors did not perform an intersectional analysis but explained the reasons for it, or they did perform the intersectional analysis, but did not include gender and provide an explanation.

**DISCUSSION**

1. **Are the findings reflected concerning sex/gender?**

*Rationale and examples:* when interpreting the results of a study, authors should evaluate the implications of sex or gender. The results should be interpreted within a gendered theoretical framework or from a gender perspective. For example, if a study finds higher rates of depression in women than in men, a gendered interpretation of data should explore gender inequalities that could explain this difference, so just reporting a gender difference would not be enough.

- **Yes:** the study met the criteria.
- **No:** the study did not meet the criteria.
- **Other (unclear, not applicable):** The reviewer is unable to determine the question.

1. **Are gender stereotypes present in the interpretation of the data?**

*Rationale and examples:* Stereotypes reflect general expectations about members of particular social groups. A gender stereotype is a generalized view or preconception about attributes or characteristics, or the roles of women and men (5). For example, understanding the higher amount of care-taking hours that women spend as a natural role based on their increased warmth and commonality.

- **Yes: t**he study met the criteria.
- **No:** the study did not meet the criteria.
- **Other (unclear, not applicable):** The reviewer is unable to determine the question or considers that it does not apply to the paper.

1. **Are there any inadequate generalizations in the study?**

*Rationale and examples:* Incorrectly assuming equality between women and men can give rise to gender bias. According to the SAGER guidelines, in the interpretation of data, the extent to which the findings can be generalized should be evaluated. For example, if the study sample is overrepresented by men (e.g. 80%), the generalization of results to women should be cautious, as it could include a systematic gender bias.

- **Yes**: the study made ANY inadequate generalization respecting sex/gender.
- **No:** the study did not make any inadequate generalizations.
- **Other: e.g** The reviewer is unable to determine the question or considers that is does not apply to the paper.

**appraisal**

Focusing on the questions formulated above, the reviewer should evaluate the gender sensitivity of each of the sections and establish an overall evaluation. Questions are not meant to be counted, but rather to guide the reviewer in the appraisal of the complete paper to establish the level of gender sensitivity,

An excellent gender-sensitive paper translates into research that includes gender in most of the research sections, provides adequate definitions of sex/gender and uses the terms correctly, evaluates bibliography concerning gender, includes gender in the main objectives, and analyzes results within a gender theoretical framework. A poor appraisal, on the contrary, means that gender was not taken into account in most of the questions presented above: e.g. sex/gender was not defined, aggregated data, results were not interpreted with respect to sex/gender. The categories of good or intermediate gender sensitivity papers are meant to be papers in between that take into account gender to some extent but still have critical gaps; e.g. mention sex/gender but use the terms interchangeably, review bibliography with respect to gender but do not provide disaggregated data.

### Apendix 4: Inter rater analysis:

Table 1: 18 reviews of studys including the word gender in the title or abstract

| Item | kappa | Standard error | p` |
| --- | --- | --- | --- |
| 1 | * | * | * |
| 2 | * | * | * |
| 3 | 1.000 | 0.000 | 0.000 |
| 4 | * | * | * |
| 5 | * | * | * |
| 6 | 0.648 | 0.183 | 0.007 |
| 7 | 0.570 | 0.163 | 0.003 |
| 8 | * | * | * |
| 9 | 0.646 | 0.319 | 0.000 |
| 10 | 0.362 | 0.261 | 0.002 |
| 11 | * | * | * |
| 12 | 0.611 | 0.188 | 0.006 |
| 13 | 0.585 | 0.151 | 0.002 |
| 14 | 0.438 | 0.200 | 0.022 |
| 15 | 0.696 | 0.186 | 0.00 |
| 16 | 0.769 | 0.150 | 0.001 |
| 17 | 0.647 | 0.318 | 0.000 |
| 18 | 1.00 | 0.000 | 0.000 |
| General Principes | 0.179 | 0.138 | 0.292 |
| Introduction | 0.630 | 0.154 | 0.000 |
| Methods | 0.244 | 0.261 | 0.156 |
| Results/Analysis | 0.523 | 0.157 | 0.000 |
| Discussion | 0.500 | 0.150 | 0.001 |
| Overal Appraisal | 0.577 | 0.146 | 0.000 |

* : kappa could not be calculated for being a constant in one or both of the raters.

### Apendix 5: List of included articles

|  | Country | Study design | % of women | Correct Use of sex/gender | Sex/gender in the literature review | Sex/Gender in the objectives | Non-binary category in data collection | Disaggregated outcome data | Sex/Gender in statistics | Gender theory in the discussion | Gender stereotypes | Overall Appraisal |
| --- | --- | --- | --- | --- | --- | --- | --- | --- | --- | --- | --- | --- |
| Bettinsoli Et al (2020) | Italy | Cross-Sectional | 40.0 | No | Yes | Yes | No | Only one of the outcomes | Factor in the regression  Interaction analysis | Yes. Differences in reported distress may be explained by gender roles and expectations. | No | Good |
| López-Atanes et al (2021) | Spain | Cross-sectional | 74.6 | No | Yes | Yes | No | Yes | Group comparison  Factor in regression  Interaction analysis | Yes. Mention to the double burden in women and its relation to mental health. | No | Good |
| Moreno-Mulet et al (2021) | Spain | Mixed-methods | 81.1 | No | Yes | Yes | No | Yes | Group comparison | Yes. Mention to gender roles and expectations. | No | Good |
| Collantoni et al (2021) | Italy | Cross-Sectional | 75.8 | No | Yes | No | No | Yes | Factor in the regression | Yes; only mention to women`s caregiver role. | No | Fair |
| Roberts et al (2021) | International | Prospective Cohort | 51.0 | No | No | No | Yes | No | Factor in the regression | No | No | Fair |
| Erquicia et al (2020) | Spain | Cross-sectional | 73.6 | No | Yes | No | No | Yes | Factor in regression  Interaction analysis | No | No | Fair |
| Carmassi et al (2021) | Italy | Cross-Sectional | 68.3 | No | Yes | No | No | Yes | Factor in the regression | No | No | Fair |
| Fattori et al (2021) | Italy | Longitudinal | 46.0 | No | No | No | No | Yes | Factor in the regression | No | No | Fair |
| Carmasi et al (2021) | Italy | Cross-sectional | 56.8 | No | Yes | Yes | No | Yes | Factor in regression  Interaction analysis | No | No | Fair |
| Forner-Puntonet et al (2021) | Spain | Intervention study | 76.6 | No | Yes | No | No | No | Group comparison | Yes. Mention to the gender perspective in mental health research. | No | Fair |
| Gago-Valiente et al (2021) | Spain | Cross- sectional | 84.9 | No | No | No | No | Yes | Group comparison | Yes. Mention to gender related variables and their influence in mental health. | No | Fair |
| Malinowska-Lipién et al (2021) | Poland | Cross-Sectional | 97.0 | Yes | No | No | No | Yes | Group comparison | Yes. Gender segregation of labor in the healthcare sector may explain poorer mental health in professions such as nursing | No | Fair |
| Weseman et al (2021) | Germany | Cross-sectional | 67.0 | No | Yes | Yes | No | No | Regression analysis | Yes. Mention to the double burden in women and its relation to mental health. | No | Fair |
| Morawa et al. (2021) | Germany | Cross-sectional | 74.8 | No | Yes | No | Yes | No | Factor in the regression | No | No | Fair |
| Mortier et al (2020) | Spain | Prospective cohort | 77.3 | No | No | No | Yes | Yes | Group comparison  Factor in the regression | No | No | Fair |
| Aguglia et al (2021) | Italy | Cross-sectional | 67.6 | No | No | No | No | No | Group comparison | No | No | Poor |
| Ali et al (2020) | Ireland | Prospective cohort | 69.0 | No | No | No | No | No | FALTA | No | No | Poor |
| Beneria et al (2020) | Spain | Cross-sectional | 70.0 | No | No | No | No | No | Group comparison  Factor in Regression | No | Yes. | Poor |
| Bidzan et al (2020) | Poland | Cross-sectional | 74.4 | No | Yes | No | No | No | Group comparison  Factor in regression | No | No | Poor |
| Di Giuseppe et al (2021) | Italy | Cross-sectional | 62.0 | No | Yes | No | No | No | Group comparison  Factor in regression | No | No | Poor |
| Dionisi et al (2021) | Italy | Longitudinal | 49.0 | No | No | No | No | No | Not analyzed | No | No | Poor |
| Ghio et al (2021) | Italy | Cross-sectional | 76.0 | No | Yes | No | No | No | Factor in regression | No | No | Poor |
| Giusti et al (2020) | Italy | Cross-sectional | 62.6 | No | No | No | No | No | Factor in regression | No | No | Poor |
| Haravuori et al (2020) | Finland | Prospective cohort | 87.5 | No | No | No | Yes, “other or prefer not to say” | No | Factor in regression | No | No | Poor |
| Lamiani et al (2021) | Italy | Cross-sectional | 80.0 | No | No | No | No | No | Factor in regression | No | No | Poor |
| Lasalvia et al (2021) | Italy | Cross-sectional | 75.3 | No | No | No | No | Yes | Group comparison  Factor in regression | No | No | Poor |
| Lucas et al (2021) | France | Cross-sectional | 79.2 | No | Yes | No | No | No | Factor in regression | No | No | Poor |
| Magnavita et al (2021) | Italy | Prospective cohort | 51.7 | No | No | No | No | No | Factor in regression | No | No | Poor |
| Man et al (2020) | Romania | Cross-sectional | 88.7 | No | No | No | No | No | Group comparison | No | No | Poor |
| Matarazzo et al (2021) | Italy | Cross-sectional | 80.2 | No | No | No | No | Yes | Group comparison  Factor in regression | No | No | Poor |
| Pappa et al (2021) | International | Cross-sectional | 68.8 | No | Yes | No | No | Yes | Group comparison  Factor in regression | No | No | Poor |
| Secosan et al (2020) | Romania | Cross-Sectional | 64.3 | No | No | No | No | No | Not analyzed | No | No | Poor |
| Secosan et al (2020) | Romania | Cross-Sectional | 64.3 | No | No | No | No | No | Not analyzed | No | No | Poor |
| Stocchetti et al (2021) | Italy | Cross-sectional | 59.0 | No | No | No | No | No | Group comparison  Factor in regression | No | No | Poor |
| Tiete et al (2021) | Belgium | Mixed-Methods | 78.4 | No | No | No | No | Yes | Group comparison  Factor in regression | No | No | Poor |
| Tselebis et al (2020) | Greece | Cross-sectional | 80.0 | No | No | No | No | Yes | Group comparison | No | No | Poor |
| Ungureanu et al (2020) | Romania | Cross-sectional | 59.4 | No | No | No | No | No | Group comparison | No | No | Poor |
| Van der Goot et al (2021) | Netherlands | Mixed-Methods | 78.3 | Yes | No | No | No | No | Not analyzed | No | No | Poor |
| Zerbini et al (2020) | Germany | Cross-sectional | 70.0 | No | No | No | No | No | Not analyzed | No | No | Poor |
| Altmayer et al (2020) | France | Cross-sectional | 78.0 | Yes | No | No | No | No | Not analyzed | No | No | Poor |
| Marcomini et al (2021) | Italy | Cross-sectional | 76.3 | No | Yes | No | No | No | Factor in regression | No | No | Poor |
| Roberts et al (2021) | International | Cross-sectional | 49.5 | No | No | No | Yes | No | Group comparison | No | No | Poor |
| Azoylay et al (2021) | France | Cross-sectional | 67.5 | No | No | No | No | No | Factor in regression | No | No | Poor |
| Mattila et al (2021) | Finnland | Cross-sectional | 87.0 | No | No | No | No | Yes | Group comparison  Factor in regression | No | No | Poor |
| Simonetti et al (2021) | Italy | Cross-sectional | 66.0 | No | Yes | No | No | Yes | Group comparison  Factor in regression | No | Yes. Higher levels of self-efficacy in men justified by “their ability to solve problems” | Poor |
| Azoulay et al (2020) | France | Cross-sectional | 71.0 | Yes | Yes | No | No | No | Group comparison  Factor in regression | No | No | Poor |
| Salopek-Žiha et al (2020) | Croatia | Cross-Sectional | Not reported | No | No | No | No | No | Not analyzed | No | No | Poor |
| Laurent et al (2021) | France | Cross-sectional | 72.6 | No | No | No | No | No | Group comparison  Factor in the analysis | Yes. Mention to the double burden of women. | No | Poor |
| González-Plaza et al (2021) | Spain | Cross-sectional | 87.0 | No | No | No | No | Yes | Not analyzed | No | No | Poor |
| Heesakkers et al (2021) | Netherlands | Cross-sectional | 73.8 | No | No | No | No | No | Factor in analysis | No | No | Poor |
| Fiabane et al (2021) | Italy | Cross-sectional | 68.2 | No | Yes | No | No | Yes | Group comparison  Factor in regression | No, but mention to the higher proportion of women in the nursing profession. | No | Poor |
| Hesselink et al (2021) | Netherlands | Cross-sectional | 72.9 | No | No | No | No | No | No | No | No | Poor |
| Ortells Abuye et al (2021) | Spain | Cross-sectional | 81.9 | No | No | No | No | No | Group comparison | No | No | Poor |
| Azoulay et al (2020) | International | Cross-sectional | 34.2 | No | No | No | No | No | Factor in the regression | No | No | Poor |
| Caillet et al (2020) | France | Cross-sectional | 75.0 | No | No | No | No | Yes | Group comparison  Factor in the regression | No | No | Poor |
| Costa et al (2021) | Italy | Cross-sectional | 52.9 | No | No | No | No | No | Group comparison  Factor in the regression | No | No | Poor |
| Diomidus et al (2020) | Greece | Cross-sectional | 71.3 | No | Yes | No | No | No | No | No | No | Poor |
| Fari et al (2021) | Italy | Retrospective cohort | 67.6 | No | No | No | No | No | Factor in the regression | No | No | Poor |
| Ilias et al (2021) | Greece | Cross-sectional | 77.0 | No | No | No | No | No | Factor in the regression | No | No | Poor |
| Kapetanos et al (2021) | Cyprus | Cross-Sectional | 80.0 | No | No | No | No | No | Factor in the regression | No | No | Poor |
| Laukkala et al (2021) | Finland | Prospective cohort | 89.0 | Yes | No | No | No | No | Group comparison  Factor in the regression | No | No | Poor |
| Leira-Sanmartin et al (2021) | Spain | Cross-sectional | 79.1 | No | No | No | No | Yes | Group comparison  Factor in the regression | No | Yes | Poor |
| Magnavita et al (2021) | Italy | Cross-sectional | 61.2 | No | No | No | No | No | Group comparison  Factor in the regression | No | No | Poor |
| Magnavita et al (2020) | Italy | Cross-sectional | 52.2 | No | No | No | No | No | Group comparison  Factor in the regression | No | No | Poor |
| Moreno-Jiménez et al (2021) | Spain | Cross-sectional | 78.7 | No | No | No | No | Yes | Factor in the regression | No | No | Poor |
| Mathijs Nijland et al (2021) | Netherlands | Intervention Study | 83.0 | No | No | No | No | Yes | Group comparison | No | No | Poor |
| Peñacoba et al (2021) | Spain | Cross-sectional | 84.8 | Yes | No | No | No | Yes | Group comparison  Factor in regression not known | No | No | Poor |
| Schmidt et al (2021) | Germany | Longitudinal | 44.8 | No | No | No | No | No | Group comparison  Factor in the regression | No | No | Poor |
| Singh et al. (2020) | Sweden | Mixed methods | 100% | Not applicable | No | No | No | No | No | No | No | Poor |
| Sangrá et al (2021) | Spain | Cross-Sectional | 84.8 | No | No | No | No | Yes | Group Comparison  Factor in the regression | No | No | Poor |
| Vanni et al (2020) | Italy | Cross-sectional | 65.2 | No | No | No | No | Yes | Group comparison | No | No | Poor |
| Van Steekiste et al |  |  |  |  |  |  |  |  |  |  |  |  |

### Apendix 6: List of excluded articles:

| Author year title of the article | Reason for Exclusion |
| --- | --- |
| Abdellah et al, 2021 - Prevalence of Poor Sleep Quality Among Physicians During the COVID-19 Pandemic | Non Europe |
| Abdoli et al, 2021 - Sources of Sleep Disturbances and Psychological Strain for Hospital Staff Working during the COVID-19 Pandemic | Wrong population |
| Ahmed et al, 2021 - Stress Levels among healthcare professionals during COVID 19 crisis response in a tertiary Cardiothoracic unit | Non accesible |
| Akman et al, 2021 - The analysis of emergency medicine professionals' occupational anxiety during the covid-19 pandemic | Non Europe |
| Altinbilek et al. 2021 - Covid-19 adversely affects the psychological status of healthcare workers in the emergency room | Non Europe |
| American Nurse. 2020 - Half of frontline nurses emotionally overwhelmed by COVID-19 | Wrong publication type |
| Arca et al, 2021 - The effect of the COVID-19 Pandemic on anxiety, depression, and musculoskeletal system complaints in healthcare workers | Non Europe |
| Arslan et al, 2021 - The effects of the COVID-19 outbreak on physicians' psychological resilience levels | Non Europe |
| Askin Ceran et al. 2021- Determination of the effect of COVID-19 pandemic on the anxiety levels and life quality of healthcare workers | Non Europe |
| Babicki et al, 2021 - The Mental Well-Being of Health Care Workers during the Peak of the COVID-19 Pandemic-A Nationwide Study in Poland | Wrong population |
| Banerjee et al, 2021 - The impact of COVID-19 on oncology professionals: results of the ESMO Resilience Task Force survey collaboration | Wrong Population |
| Bassi et al, 2021 - The relationship between post-traumatic stress and positive mental health symptoms among health workers during COVID-19 pandemic in Lombardy, Italy | Wrong population |
| Bates et al, 2020 - Psychological impact of caring for critically ill patients during the Covid-19 pandemic and recommendations for staff support | Non Europe |
| Bhargava et al. 2020 - Mental distress in dermatologists during COVID-19 pandemic: Assessment and risk factors in a global, cross-sectional study | Non validated scale |
| Boktor et al, 2020 - Stress and Anxiety Management During the COVID-19 Pandemic (Lessons Learnt from a Cohort of Orthopaedic Registrars Redeployed to ITU) | Non Europe |
| Bosco et al., 2021 - Challenges to the orthopedic resident workforce during the first wave of COVID-19 pandemic: Lessons learnt from a global cross-sectional survey | Wrong Outcome (not mental health) |
| Brejnebol et al, 2021 - Stress reactions in a tertiary oto-rhino-laryngological department during the first wave of the COVID-19 pandemic in the Danish Capital region | Non accesible |
| Bruffaerts et al., 2021 - Suicidality among healthcare professionals during the first COVID19 wave | Wrong Population |
| Caillet et al. 2021 - Intensive Care Nurses, Psychological Disorders and COVID-19. The COVID IMPACT NATIONAL STUDY | Foreign Language |
| Cakmak et al. 2021 - Being a caregiver in the palliative care unit in the pandemic: who can do it better? | Non Europe |
| Cakmak et al. 2021 - Being Both a Parent and a Healthcare Worker in the Pandemic: Who Could Be Exhausted More? | Non Europe |
| Carmassi et al, 2021 - Post-traumatic stress disorder, burnout and their impact on global functioning in Italian emergency healthcare workers | Wrong Population |
| Carmassi et al. 2021 - Mental health of frontline help-seeking healthcare workers during the COVID-19 outbreak in the first affected hospital in Lombardy, Italy | Wrong publication type |
| Chan et al, 2021 - Psychological Impact of the COVID-19 Pandemic on Licensed Full-Time Practicing Nurses Undertaking Part-Time Studies in Higher Education: A Cross-Sectional Study | Non Europe |
| Chapa-Koloffon. 2021 - Frequency of acute stress disorder in health care workers of a tertiary level pediatric hospital during the National Safe Distance Strategy for COVID-19 prevention | Non Europe |
| Chen et al. 2021 A Large-Scale Survey on Trauma, Burnout, and Posttraumatic Growth among Nurses during the COVID-19 Pandemic | Non Europe |
| Chou et al, 2021 - Otolaryngology Resident Wellness, Training, and Education in the Early Phase of the COVID-19 Pandemic | Non Europe |
| Chu et al. 2021 - Hospital-Based Health Care Worker Perceptions of Personal Risk Related to COVID-19 | Non Europe |
| Cinaroglu et al. 2021 - Field study for determining the effect of covid-19 on healthcare workers effect of covid-19 on healthcare workers | Non Europe |
| Coco et al, 2021 - Psychosocial Impact and Role of Resilience on Healthcare Workers during COVID-19 Pandemic | Wrong Population |
| Constantini et al. 2021 - COVID-19 pandemic distress among a sample of Italian psycho-oncologists: risk of isolation and loneliness | Wrong population |
| Coto et al, 2020 - The impact of COVID-19 on allied health professions | Non Europe |
| Csigo et al. 2021 - First Reactions and Attitudes of Psychiatric Workers in Budapest Psychiatric Care Units Regarding the COVID-19 Pandemic | Non validated scale |
| D'emeh et al, 2021 - Work-Related Stress and Anxiety Among Frontline Nurses During the COVID-19 Pandemic A Cross-Sectional Study | Non Europe |
| Dal'Bosco et al. 2020 - Mental health of nursing in coping with COVID-19 at a regional university hospital | Non Europe |
| Dantas et al. 2021 - Factors associated with anxiety in multiprofessional health care residents during the COVID-19 pandemic | Non Europe |
| De Sio et al., 2020 - The impact of COVID-19 on doctors' well-being: results of a web survey during the lockdown in Italy | Wrong Population |
| Demerdash et al. 2021 - Evaluation of copeptin and psychological stress among healthcare providers during COVID-19 pandem | Non Europe |
| Denning et al. 2021 - Determinants of burnout and other aspects of psychological well-being in healthcare workers during the COVID-19 pandemic: A multinational cross-sectional study | Wrong Population |
| Dharra et al, 2021 - Promoting Mental Health of Nurses During the Coronavirus Pandemic: Will the Rapid Deployment of Nurses' Training Programs During COVID-19 Improve Self-Efficacy and Reduce Anxiety? | Non Europe |
| Dikes, N. 2021. Assessing the psychological impact of COVID-19 on intensive care workers: A single-centre cross-sectional UK-based study | Non Europe |
| Doo et al. 2021 - Influence of anxiety and resilience on depression among hospital nurses: A comparison of nurses working with confirmed and suspected patients in the COVID-19 and non-COVID-19 units | Non Europe |
| Dyer et al. 2021 - Evaluation of a reiki program for healthcare workers negatively impacted by the pandemic | Wrong publication type |
| Elbay et al. 2020 - Depression, anxiety, stress levels of physicians and associated factors in Covid-19 pandemics | Non Europe |
| Elhadi et al, 2020 - The Mental Well-Being of Frontline Physicians Working in Civil Wars Under Coronavirus Disease 2019 Pandemic Conditions | Non Europe |
| Emmanuel et al, 2021 - Study on effectiveness of staff welfare program regarding occupational stress during covid 19 pandemic among nursing officers | Non Europe |
| Ezzat et al., 2021 - The global mental health burden of COVID-19 on critical care staff | Non Europe |
| Fauzi et al. 2020 - Doctors' Mental Health in the Midst of COVID-19 Pandemic: The Roles of Work Demands and Recovery Experiences | Non Europe |
| Ferreira et al. 2021 A Wake-up Call for Burnout in Portuguese Physicians During the COVID-19 Outbreak: National Survey Study | Wrong population |
| Fino et al. 2021 - Helping patients connect remotely with their loved ones modulates distress in healthcare workers: a tend-and-befriend hypothesis for COVID-19 front liners | Wrong publication type |
| Flateau et al, 2021 - Psychological impact of the SARS-CoV-2 outbreak on the staff of a French hospital | Wrong Population |
| Fleuren et al., 2021 - We're Not Gonna Fall: Depressive Complaints, Personal Resilience, Team Social Climate, and Worries about Infections among Hospital Workers during a Pandemic | Non validated scale |
| Florin et al, 2020- Socio-economic and psychological impact of the COVID-19 outbreak on private practice and public hospital radiologists | Wrong population |
| Fournier et al, 2021 - Positive reappraisal coping strategies among intensive care professionals in France in response to the COVID-19 pandemic | Non accesible |
| Fournier et al. 2021- Availability and use of supports during the COVID-19 health crisis | Non accesible |
| Fteroupolli et al. 2021 - Beyond the physical risk: Psychosocial impact and coping in healthcare professionals during the COVID-19 pandemic | Wrong population |
| García-Hedrera et al. 2021 - Intensive care unit professionals during the COVID-19 pandemic in Spain: social and work-related variables, COVID-19 symptoms, worries, and generalized anxiety levels | Wrong Population |
| Ghaleb et al. 2021 - Mental health impacts of COVID-19 on healthcare workers in the Eastern Mediterranean Region: a multi-country study | Non Europe |
| Ghislieri et al, 2021 - Work-family conflict during the Covid-19 pandemic: teleworking of administrative and technical staff in healthcare. An Italian study | Wrong population |
| Giannouli et al, 2021 - The Flight of Icarus: A Preliminary Study of the Emotional Correlates of Hubris in Gerontological Nurses during the SARS-CoV-2 Pandemic | Wrong population |
| Giménez Beltran et al. 2021. Burn out syndrome in ICU workers amidst covid-19 pandemic | Non accesible |
| Gonzalez-Pando et al., 2021 - The role of nurses' professional values during the COVID-19 crisis | Wrong Population |
| Gormez et al. 2021 - Has COVID-19 taken a heavier toll on the mental health of ICU nurses? | Wrong publication type |
| Guha et al, 2021 - Not Just a 'Breath of Death': Indirect Consequences of Working in a COVID-19 Unit | Wrong Population |
| Guillen-Astete et al. 2020 - Levels of anxiety and depression among emergency physicians in Madrid during the SARS-CoV-2 pandemic | Wrong Population |
| Gul et al. 2021 - Determining anxiety levels and related factors in operating room nurses during the COVID-19 pandemic: A descriptive study | Non Europe |
| Harry, 2021 - Predictors of burnout for frontline nurses in the COVID-19 pandemic: Well-being, satisfaction with life, social support, fear, work setting factors, psychological impacts, and self-efficacy for nursing tasks | Wrong publication type |
| Hassamal et al, 2021 - The Psychological Impact of COVID-19 on Hospital Staff | Non Europe |
| He et al, 2021 -Risk Factors for Anxiety and Depressive Symptoms in Doctors During the Coronavirus Disease 2019 Pandemic | Non Europe |
| Hennei et al, 2021 - Racial and Gender Discrimination Predict Mental Health Outcomes among Healthcare Workers Beyond Pandemic-Related Stressors: Findings from a Cross-Sectional Survey | Non Europe |
| Hilmi et al, 2020 - Professional and Psychological Impacts of the COVID-19 Pandemic on Oncology Residents: A National Survey | Wrong Population |
| Hines et al, 2021 - Trends in Moral Injury, Distress, and Resilience Factors among Healthcare Workers at the Beginning of the COVID-19 Pandemic | Non Europe |
| Hoedel et al. 2021 - Associations between personal protective equipment and nursing staff stress during the COVID-19 pandemic | Wrong Population |
| Horn, 2021 - Psychological impact of the COVID-19 pandemic on non-frontline healthcare workers | Wrong publication type |
| Huang et al. 2020 - [Mental health survey of medical staff in a tertiary infectious disease hospital for COVID-19] | Non Europe |
| Implementation of a Mindfulness-Based Crisis Intervention for Frontline Healthcare Workers During the COVID-19 Outbreak in a Public General Hospital in Madrid, Spain | Non-validated scale |
| Jiang et al, 2021 - Psychological status of the staff in a general hospital during the outbreak of coronavirus disease 2019 and its influential factors | Non Europe |
| Kader et al, 2021 - Perceived stress and post-traumatic stress disorder symptoms among intensive care unit staff caring for severely ill coronavirus disease 2019 patients during the pandemic: a national study | Non Europe |
| Kaltiso et al, 2021 - The impact of racism on emergency health care workers | Non Europe |
| Kameg et al. 2021 - Mental wellness among psychiatric-mental health nurses during the COVID-19 pandemic | Wrong Population |
| Kameno et al. 2021 - Individual psychotherapy using psychological first aid for frontline nurses at high risk of psychological distress during the COVID-19 pandemic | Non Europe |
| Kannampallil et al. 2021 - Exposure to COVID-19 patients increases physician trainee stress and burnout | Non Europe |
| Karasu et al, 2021 - The impact of COVID-19 on healthcare workers' anxiety levels | Non Europe |
| Kaya et al, 2021 - The Effect of COVID-19 Pandemic to the Practices of Cardiology Clinics and on the Anxiety Levels of Cardiologists | Non Europe |
| Kazgan et al, 2021 - The relationship between hope in healthcare employees and social support and coping ability during the outbreak process: Hope in healthcare employees | Non Europe |
| Kelker et al. 2021 - Longitudinal Prospective Study of Emergency Medicine Provider Wellness Across Ten Academic and Community Hospitals During the Initial Surge of the COVID-19 Pandemic | Non Europe |
| Khajuria et al, 2021 - Workplace factors associated with mental health of healthcare workers during the COVID-19 pandemic: an international cross-sectional study | Wrong Population |
| Khani et al. 2021 - Healthcare workers experience of the Covid19 pandemic: Perspectives from the frontline | Wrong publication type |
| Khattab et al, 2020 - The short-term impact of COVID-19 pandemic on spine surgeons: a cross-sectional global study | Non Europe |
| Kirk et al, 2021 - Psychosocial impact of the COVID-19 pandemic on paediatric healthcare workers | Non Europe |
| Kolodziej et al, 2021 - The impact of the COVID-19 pandemic on Polish orthopedics, in particular on the level of stress among orthopedic surgeons and the education process | Non validated scale |
| Kramer et al, 2021 - Subjective burden and perspectives of German healthcare workers during the COVID-19 pandemic | Wrong Population |
| Krammer et al. 2020- Adjustment Disorder, Depression, Stress Symptoms, Corona Related Anxieties and Coping Strategies during the Corona Pandemic (COVID-19) in Swiss Medical Staff | Non Europe |
| Kristoffersen et al., 2021 - Experiences, distress and burden among neurologists in Norway during the COVID-19 pandemic | Non Europe |
| Krok et al, 2021 - Risk of contracting covid-19, personal resources and subjective well-being among healthcare workers: The mediating role of stress and meaning-making | Wrong Population |
| Krupa et al, 2021 - Sleep disorders among nurses and other health care workers in Poland during the COVID-19 pandemic | Wrong Population |
| Kuki et al. 2021 - Effects of contact with COVID-19 patients on the mental health of workers in a psychiatric hospital | Non Europe |
| Layek et al, 2021 -Self-isolation of healthcare workers during covid-19 pandemic in a tertiary care center - association between their sleep quality, anxiety status and social capital | Non Europe |
| Le et al, 2021 - Psychological Effects of Screen Time in Health Care Workers During the COVID-19 Pandemic | Non Europe |
| Lee et al, 2021 - Risk Perception, Unhealthy Behavior, and Anxiety Due to Viral Epidemic Among Healthcare Workers: The Relationships With Depressive and Insomnia Symptoms During COVID-19 | Non Europe |
| Lin et al. 2021 - COVID-19 Pandemic Is Associated with an Adverse Impact on Burnout and Mood Disorder in Healthcare Professionals | Non Europe |
| Liu et al, 2020 - Psychological impact in non-infectious disease specialists who had direct contact with patients with COVID-19 | Non Europe |
| Liu et al., 2020 - Psychological impact in non-infectious disease specialists who had direct contact with patients with COVID-19 | Non Europe |
| Lixia et al. 2021 - A cross-sectional study of the psychological status of 33,706 hospital workers at the late stage of the COVID-19 outbreak | Non Europe |
| Lovell-Viggers et al. 2021- COVID-19 pandemic and the hidden front line | Wrong publication type |
| Luceno-Moreno et al, 2020 - Symptoms of Posttraumatic Stress, Anxiety, Depression, Levels of Resilience and Burnout in Spanish Health Personnel during the COVID-19 Pandemic | Wrong Population |
| Ma et al, 2021 - Psychological stress among health care professionals during the 2019 novel coronavirus disease Outbreak: Cases from online consulting customers | Non Europe |
| Marinaci et al. 2020 Emotional distress, psychosomatic symptoms and their relationship with institutional responses: A survey of Italian frontline medical staff during the Covid-19 pandemic | Wrong Population |
| Matsumoto et al. 2021 - Factors affecting mental illness and social stress in hospital workers treating COVID-19: Paradoxical distress during pandemic era | Non Europe |
| Maunder et al, 2021 - Psychological impact of the COVID-19 pandemic on hospital workers over time: Relationship to occupational role, living with children and elders, and modifiable factors | Non Europe |
| Mcall et al, 2021 - Short-term insomnia disorder in health care workers in an academic medical center before and during COVID-19: rates and predictive factors | Non Europe |
| Meo et al. 2021 Comparison of Generalized Anxiety and Sleep Disturbance among Frontline and Second-Line Healthcare Workers during the COVID-19 Pandemic | Non Europe |
| Miguel-Puga et al. 2021 - Burnout, depersonalization, and anxiety contribute to post-traumatic stress in frontline health workers at COVID-19 patient care, a follow-up study | Non Europe |
| Mira et al, 2020 - Preventing and Addressing the Stress Reactions of Health Care Workers Caring for Patients With COVID-19: Development of a Digital Platform (Be + Against COVID) | Wrong Outcome (not mental health) |
| Mishra et al. 2021 - Comparison of Psychological Morbidity of Health Care Workers Posted in COVID and Non COVID Labour Rooms | Non Europe |
| Mounir et al, 2021 - Psychological Distress and Tobacco Use Among Hospital Workers During COVID-19 | Non validated scale |
| Murat et al. 2021 Determination of stress, depression and burnout levels of front-line nurses during the COVID-19 pandemic | Non Europe |
| Neff et al, 2021 - [Psychological impact of involvement of medical and psychological emergency unit professionals in the medical and psychological care system of the COVID-19 epidemic] | Foreign Language |
| Nie et al, 2020 - Psychological impact of COVID-19 outbreak on frontline nurses: A cross-sectional survey study | Non Europe |
| Nourian et al, 2021 - The Impact of an Online Mindfulness-Based Stress Reduction Program on Sleep Quality of Nurses Working in COVID-19 Care Units: A Clinical Trial | Non Europe |
| Onen Sertoz et al. 2021 - Burnout in Healthcare Professionals During the Covid-19 Pandemic in a Tertiary Care University Hospital: Evaluation of the Need for Psychological Support | Non Europe |
| Orru et al, 2021 - Secondary Traumatic Stress and Burnout in Healthcare Workers during COVID-19 Outbreak | Wrong Population |
| Ou et al, 2021 - Resilience of nurses in isolation wards during the COVID⁃19 pandemic: a cross-sectional study | Non Europe |
| Ozen et al. 2021 - Comparison of emotional approaches of medical doctors against COVID-19 pandemic: Eastern and Western Mediterranean countries | Wrong Population |
| Park et al. 2020 - COVID-19 Outbreak and Its Association with Healthcare Workers' Emotional Stress: a Cross-Sectional Study | Non Europe |
| Patel et al, 2021 - Study of correlates of depression among health care workers during COVID-19 epidemic | Non Europe |
| Pérez-Ortega et al, 2020 - Situación de la enfermera de cardiología durante la pandemia COVID-19 | Wrong Population |
| Poncelet et al. 2021 - Job stress in paediatric ICU staff caring for adult COVID-19 patients: An observational study during the first COVID-19 wave | Wrong publication type |
| Putrino et al. 2020 - Multisensory, Nature-Inspired Recharge Rooms Yield Short-Term Reductions in Perceived Stress Among Frontline Healthcare Workers | Non Europe |
| Rajcani et al, 2021 - Stress and hair cortisol concentrations in nurses during the first wave of the COVID-19 pandemic | Wrong Population |
| Ranieri et al, 2021 - Prolonged COVID 19 Outbreak and Psychological Response of Nurses in Italian Healthcare System: Cross-Sectional Study | Wrong Population |
| Rodriguez-Vega et al. 2020 - Implementation of a Mindfulness-Based Crisis Intervention for Frontline Healthcare Workers During the COVID-19 Outbreak in a Public General Hospital in Madrid, Spain | Non validated scale |
| Rossi et al. 2020 Mental Health Outcomes Among Frontline and Second-Line Health Care Workers During the Coronavirus Disease 2019 (COVID-19) Pandemic in Italy | Wrong Population |
| Saracoglu et al, 2021 - The Psychological Impact of COVID-19 Disease is more Severe on Intensive Care Unit Healthcare Providers: A Cross-sectional Study | Non Europe |
| Seow et al, 2020 - Stress and Strain in an Orthopaedic Department on the Frontlines during the COVID-19 Pandemic: An Analysis of Burnout and the Factors Influencing It | Foreign Language |
| Shahzad et al, 2020- Perceived Threat of COVID-19 Contagion and Frontline Paramedics' Agonistic Behaviour: Employing a Stressor-Strain-Outcome Perspective | Non Europe |
| Shalhub et al. 2021 - Global vascular surgeons' experience, stressors, and coping during the coronavirus disease 2019 pandemic | Non Europe |
| Sharma et al. 2020 A cross-sectional analysis of prevalence and factors related to depression, anxiety, and stress in health care workers amidst the COVID-19 pandemic | Non Europe |
| Shayganfard et al, 2021 - Sources of Health Anxiety for Hospital Staff Working during the Covid-19 Pandemic | Non Europe |
| Shen et al, 2020 - Psychological stress of ICU nurses in the time of COVID-19 | Non Europe |
| Shresta et al, 2020 - Prevalence of Psychological Effect of COVID-19 on Medical Professionals in a Tertiary Care Center | Non Europe |
| Soares et al, 2021 - The Impact of COVID-19 on Dual-Physician Couples: A Disproportionate Burden on Women Physicians | Non validated scale |
| Somville et al, 2021 - Work stress-related problems in physicians in the time of COVID-19 | Non validated scale |
| Sugihara et al. 2021 - COVID-19 vaccination and mental health in hospital workers | Wrong publication type |
| Sung. 2021 - Mental health crisis in healthcare providers in the COVID-19 pandemic: a cross-sectional facility-based survey | Non Europe |
| Tarquinio et al. 2021 - EMDR in Telemental Health Counseling for Healthcare Workers Caring for COVID-19 Patients: A Pilot Study | Wrong Population |
| Trumello et al, 2020 - Psychological Adjustment of Healthcare Workers in Italy during the COVID-19 Pandemic: Differences in Stress, Anxiety, Depression, Burnout, Secondary Trauma, and Compassion Satisfaction between Frontline and Non-Frontline Professionals | Wrong Population |
| Uyaroglu et al. 2020 - Evaluation of the effect of COVID-19 pandemic on anxiety severity of physicians working in the internal medicine department of a tertiary care hospital: a cross-sectional survey | Non Europe |
| Vitale et al, 2021 - Observational study on the potential psychological factors that affected Italian nurses involved in the COVID-19 health emergency | Wrong Population |
| Vitale et al. 2021 - Anxiety, burnout and depression levels according to sex and years of work experience in Italian nurses engaged in the care of COVID-19 patients | Wrong Population |
| Vu et al. 2021 - Impacts of Digital Healthy Diet Literacy and Healthy Eating Behavior on Fear of COVID-19, Changes in Mental Health, and Health-Related Quality of Life among Front-Line Health Care Workers | Non Europe |
| Wang et al, 2021 - The prevalence and risk factors for depressive symptoms in frontline nurses under COVID-19 pandemic based on a large cross-sectional study using the propensity score-matched method | Non Europe |
| Weilenmann et al. 2021 - Health Care Workers' Mental Health During the First Weeks of the SARS-CoV-2 Pandemic in Switzerland-A Cross-Sectional Study | Non Europe |
| Wild et al, 2021 - Post-traumatic stress disorder and major depression among frontline healthcare staff working during the COVID-19 pandemic | Non Europe |
| Wozniak et al. 2021 - Mental health outcomes of ICU and non-ICU healthcare workers during the COVID-19 outbreak: a cross-sectional study | Non Europe |
| Wu et al, 2020 - Stressors of nurses in psychiatric hospitals during the COVID-19 outbreak | Non Europe |
| Xiong et al, 2020 - The Psychological Status and Self-Efficacy of Nurses During COVID-19 Outbreak: A Cross-Sectional Survey | Non Europe |
| Yang et al, 2021 - Psychological impact of COVID-19 on hospital workers in nursing care hospitals | Non Europe |
| Yildirim et al, 2020 - Perceived Risk and Mental Health Problems among Healthcare Professionals during COVID-19 Pandemic: Exploring the Mediating Effects of Resilience and Coronavirus Fear | Non Europe |
| Yu et al, 2020 - Psychological Behavior of Frontline Medical Staff in the Use of Preventive Medication for COVID-19: A Cross-Sectional Study | Non Europe |
| Yurtseven et al. 2021. Anxiety levels of university hospital nurses during the Covid-19 pandemic | Non Europe |
| Zaghini et al. 2021 - A mixed methods study of an organization's approach to the COVID-19 health care crisis | Non validated scale |
| Zakeri et al, 2021 - The relationship between frontline nurses' psychosocial status, satisfaction with life and resilience during the prevalence of COVID-19 disease | Non Europe |
| Zuinen et al., 2020 - Psychological evaluation of the healthcare workers in a Geriatric Unit during the Covid 19 | Non accesible |

### Bibliography:

1. Hammarström A, Johansson K, Annandale E, Ahlgren C, Aléx L, Christianson M, et al. Central gender theoretical concepts in health research: The state of the art. J Epidemiol Community Health. 2014;68(2):185–90.

2. Toolkit Gender in EU-funded research.

3. Heidari S, Babor TF, De Castro P, Tort S, Curno M. Sex and Gender Equity in Research: rationale for the SAGER guidelines and recommended use. Res Integr Peer Rev. 2016;

4. Clayton JA, Tannenbaum C. Reporting sex, gender, or both in clinical research? JAMA - J Am Med Assoc. 2016;316(18):1863–4.

5. Nadal KL. Gender Stereotypes. SAGE Encycl Psychol Gend. 2017;(September 2017):1–24.
